# A dot-stripe Turing model of joint patterning in the tetrapod limb

**DOI:** 10.1242/dev.183699

**Published:** 2020-04-12

**Authors:** Jake Cornwall Scoones, Tom W. Hiscock

**Affiliations:** 1 Department of Zoology, University of Cambridge, Cambridge CB2 3EJ, UK; 2 Wellcome Trust/Cancer Research UK Gurdon Institute, University of Cambridge, Cambridge CB2 1QN, UK; 3 Cancer Research UK Cambridge Institute, University of Cambridge, Cambridge CB2 0RE, UK

**Keywords:** Limb patterning, Mathematical modelling, Turing patterns

## Abstract

Iterative joints are a hallmark of the tetrapod limb, and their positioning is a key step during limb development. Although the molecular regulation of joint formation is well studied, it remains unclear what controls the location, number and orientation (i.e. the pattern) of joints within each digit. Here, we propose the dot-stripe mechanism for joint patterning, comprising two coupled Turing systems inspired by published gene expression patterns. Our model can explain normal joint morphology in wild-type limbs, hyperphalangy in cetacean flippers, mutant phenotypes with misoriented joints and suggests a reinterpretation of the polydactylous Ichthyosaur fins as a polygonal joint lattice. By formulating a generic dot-stripe model, describing joint patterns rather than molecular joint markers, we demonstrate that the insights from the model should apply regardless of the biological specifics of the underlying mechanism, thus providing a unifying framework to interrogate joint patterning in the tetrapod limb.

## Abstract (precis)

**Summary:** A dot-stripe Turing mechanism can reproduce a wide diversity of joint patterns observed in the tetrapod lineage, providing a unifying framework to interrogate joint patterning in the tetrapod limb.

## INTRODUCTION

The acquisition of digits (fingers and toes) was a key step in the evolution of the tetrapod limb and coincided with the invasion of aquatic vertebrates onto land (Coates, 1996; Coates and Clack, 1990; Jarvik, 1996). Digits develop in the most distal part of the limb, the autopod, and form a repeating set of distally oriented, jointed skeletal rods (typically five). In general, the autopod has a highly conserved morphology, with many species exhibiting a canonical pentadactyl state (Saxena et al., 2017). However, there exists considerable variation in digit number and morphology between species, generating limbs that are well-adapted for tasks as diverse as walking, grasping, perching, flying or swimming (Kavanagh et al., 2013).

To generate these complex morphologies, the developing limb must be patterned in a number of ways. First, proximal-distal (PD) patterning separates the stylopod and zeugopod (proximal and distal long bones of the arm/leg), the mesopod (wrist/ankle) and the autopod (hand/foot), and is underpinned by a combination of cell-intrinsic programmes [e.g. collinear Hox gene activation (Zakany and Duboule, 2007)] and long-range morphogen gradients [e.g. FGFs from the apical ectodermal ridge, AER (Zuniga, 2015)]. Second, the initially homogeneous limb bud mesenchyme spontaneously breaks symmetry to form a periodic pattern, specifying digital and interdigital fates in an alternating fashion along the distal edge of the handplate (Newman and Frisch, 1979; Sheth et al., 2012). Recent work demonstrated that this digit-interdigit patterning is achieved by a Turing-like mechanism, relying on a molecular network of secreted Bmp and Wnt genes (Raspopovic et al., 2014). Third, the autopod is patterned along the anterior-posterior (AP) axis via a gradient of SHH signalling, which confers differential morphological ‘identities’ to each digit under the paradigmatic French Flag model (Huang et al., 2016; Wolpert, 1969).

Here, we focus on a fourth patterning module that is operative during late stage autopod development: joint patterning. During joint patterning, a repeated set of joints must be positioned within each of the digit rays, acting as hinges that allow the digits to flex and bend. The appearance of these repeated joint patterns in the fossil record marks the emergence of tetrapod limb morphology, exemplified in the polydactylous *Acanthostega* (Coates and Clack, 1990). In extant tetrapods, joint patterns show significant variation, ranging from the widely spaced joints enabling flight of the bat wing (Sears et al., 2006), to the hyperphalangeal flippers of whales and dolphins (Cooper et al., 2007, 2018).

A crucial step in joint formation is the specification of joint progenitors, which downregulate genes associated with the chondrogenic programme (e.g. *Sox9* and *Col2a1*) and upregulate joint markers (e.g. *Jun*, *Gdf5*, *Wnt4* and *Wnt9a*) (Guo et al., 2004; Hartmann and Tabin, 2001; Kan and Tabin, 2013; Merino et al., 1999; Sohaskey et al., 2008; Storm and Kingsley, 1999). These then differentiate into a variety of cell fates, forming the articular cartilage, ligaments, synovial fluid and fibrous capsule that constitute a mature synovial joint and divide the mature digit into phalanges. Molecular signatures of joint progenitors first emerge in the interzone, a region of elevated cell density that marks the site of future joints (Decker et al., 2014). Interzones form sequentially at the distal end of the digit ray, which lengthens over time as new cells are incorporated from a population of dividing progenitors located beneath the AER. At the same time, an incipient phalanx is also specified distally, in a region referred to as the phalanx-forming region (PFR) (Suzuki et al., 2008). An understanding of this early step in joint formation (i.e. where the interzones form) provides crucial insight into joint patterning and thus digit morphology. In this work, we aim both to describe and explain three key aspects of joint patterning: the location, the number and the orientation of joints within each digit.

Multiple molecular pathways have been implicated in joint patterning. Several Wnt ligands (e.g. *Wnt4* and *Wnt9a*) are expressed in prospective joints, and WNT/β-catenin signalling is necessary and sufficient to initiate the joint programme (Guo et al., 2004). *Gdf5* is also expressed in joint regions, but paradoxically inhibits the joint fate: *Gdf5*^−/−^ digits display ectopic specification of joint progenitors, whereas *Gdf5*-soaked beads locally inhibit joint formation when placed proximate to a maturing interzone (Merino et al., 1999; Storm and Kingsley, 1999). Other secreted TGFβs, such as *Bmp2*, are expressed in the interdigital mesenchyme, and multiple lines of evidence suggest that levels of BMP signalling influence joint positioning (Brunet et al., 1998; Suzuki et al., 2008). Moreover, high BMP activity is detected at the distal edge of each digit ray, which has been associated with the PFR (Suzuki et al., 2008) and digital crescent (Montero et al., 2008). BMP activity is further modulated by expression of the BMP antagonist *Noggin* in the digit ray, and by a *Shh*-regulated gradient of *5′Hoxd-Gli3* activity along the AP axis (Huang et al., 2016). *Ihh* presents an additional source of Hedgehog signalling, expressed in the centre of each phalanx, that regulates *Gdf5* expression in neighbouring interzones (Gao et al., 2009). How do these diverse molecular pathways cooperate to govern the location, number and orientation of developing joints?

Previous studies have suggested that a Turing-like mechanism may be responsible for the iterative nature of the joint pattern (Tanaka and Iber, 2013). Turing mechanisms allow homogeneous tissues to break symmetry spontaneously, forming a set of periodically arranged repeated structures (Turing, 1952). These can be a set of repeated dots, as seen in arrays of epidermal appendages such as hair follicles (Sick et al., 2006) and feather buds (Ho et al., 2019); or a set of repeated stripes, e.g. in the pigmentation stripes of the zebrafish (Nakamasu et al., 2009) and rugae of the mammalian palate (Economou et al., 2012). The Turing mechanism works by combining local activation (e.g. a short-ranged diffusible activator) with long-range inhibition (e.g. a long-ranged diffusible inhibitor), forming a network capable of self-organizing periodic patterns.

Several experiments suggest that a Turing-type system is operating within the digit. First, specification of an ectopic joint in chick digits via *Wnt9a* overexpression represses the joint formation that would have otherwise occurred in its vicinity, suggestive of long-range inhibition between joints (Hartmann and Tabin, 2001). Second, blocking signals from the proximal regions of the digit using a foil barrier perturbs distal joint positioning (Kavanagh et al., 2013). Third, the highly repetitive hyperphalangeal patterns seen in whales and dolphins, with up to 17 joints forming in a single digit, can be well explained by an intrinsically periodic Turing mechanism but is difficult to reconcile with a mechanism whereby the location of each joint is separately specified (Fedak and Hall, 2004).

Here, we aim to build a mathematical model, inspired by the Turing mechanism, that can explain joint position, number and orientation. We propose the dot-stripe mechanism for joint patterning, which combines two separate Turing systems to self-organize dots and stripes. We demonstrate that this mechanism recapitulates the spatiotemporal dynamics of patterning in wild-type limbs. It can also explain non-intuitive joint orientations observed in mutants, and unusual joint morphologies that are observed in the tetrapod lineage.

## RESULTS

### A dot-stripe mechanism for joint patterning

**Fig. 1.**
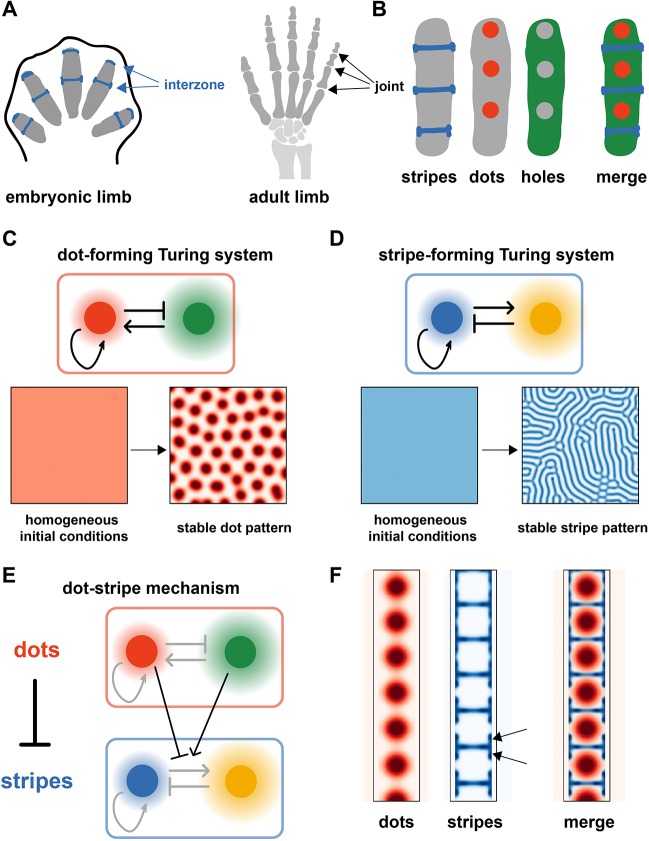
**The dot-stripe model of joint patterning.** (A) Schematic of joint patterning. (B) Summary of gene expression patterns in the developing digit (see Table S1). (C) A Turing system that spontaneously breaks symmetry to form dots (Eqn 1). (D) A different Turing system that spontaneously breaks symmetry to form stripes (Eqn 2). (E) Schematic of the coupled dot-stripe mechanism (Eqn 4). (F) Simulation of the dot-stripe model results in evenly spaced dots (red) with interspersed stripes (blue). Arrows highlight expression patterns at the outer edges of the joint.

To formulate a mathematical model for joint patterning, we began by surveying the literature for gene expression patterns that correlate with joint position, paying particular attention to genes that have a joint phenotype when knocked out or overexpressed, in either mouse or chick. We restricted our attention to genes that are expressed within the newly forming phalanx, thereby focusing on early patterning events rather than later stage joint differentiation markers [e.g. tenascin (Koyama et al., 2007)]. From this broad catalogue (see Table S1), we identified several classes of genes, which we refer to as ‘Stripe’, ‘Dot’ and ‘Hole’ (Fig. 1A,B). ‘Stripe’ genes are expressed in narrow stripes, specifying the interzones and future joint sites, and include canonical joint markers such as *Gdf5* and *Wnt* ligands (Hartmann and Tabin, 2001; Kan and Tabin, 2013; Storm and Kingsley, 1999). In contrast, ‘Dot’ genes are expressed as dots central to each phalanx, out of phase with the ‘Stripe’ genes, and include *Ihh* and activated BMP signalling effectors (*pSmad*) (Gao et al., 2009; Huang et al., 2016). Finally, ‘Hole’ genes are expressed throughout the phalanx apart from its centre, forming a hole-like pattern complementary to the ‘Dot’ pattern (e.g. *Hip1* and *Gli1*) (Gao et al., 2009).

Based on these observations, we posited a dot-stripe model for joint patterning, whereby joints are formed as narrow stripes, with their location determined by repression from a set of repeating dots. In this model, there are two key components: patterning of a series of dots that ultimately set joint position; and subsequent refinement of this pattern to form straight, stable and narrow stripes that divide the mature digit into phalanges.

To formalize this hypothesis, we constructed a mathematical model of joint patterning. One approach would be to base the model on known molecular interactions, directly modelling the important signalling molecules (e.g. Wnts, Gdf5 and Ihh), transcription factors (e.g. Sox9 and Jun) and their interactions [as has been done for digit-interdigit patterning (Raspopovic et al., 2014)]. However, although many of the important genes have been identified, the interactions between them remain poorly characterized, making it difficult to construct a comprehensive model. We therefore adopt an alternative modelling strategy that aims to capture the phenomenology of patterning, while remaining agnostic to the specific molecular mechanism. Recent work has suggested that Turing systems are rather generic, in that they can be formed from more realistic and complicated molecular circuits (Marcon et al., 2016), and can incorporate non-molecular processes such as cell migration and mechanical forces, while retaining the same qualitative dynamics (Hiscock and Megason, 2015a). Therefore, one can model many qualitative features of Turing systems using simpler, canonical models.

Applying these principles to joint patterning, we first sought a mechanism to generate evenly spaced dots: we use a canonical, two-node Turing network, termed the activator-substrate model (Gierer and Meinhardt, 1972). The two components of the model form dot- and hole-like patterns, respectively, mirroring the spatial localization of the ‘Dot’ and ‘Hole’ genes (Fig. 1C, Fig. S1A). The second part of the model must generate stripes. It is well known that a Turing mechanism can form stripes instead of dots, provided the parameters of reaction rates and diffusion constants are in a certain range; here, we use the canonical activator-inhibitor Turing circuit (Fig. 1D, Fig. S1B). In this case, we can assign putative identities to the genes: several Wnt ligands fulfil the requirements for the activator molecule (they are expressed in joints and promote joints), whereas *Gdf5* satisfies the requirements for the inhibitor (it is expressed in joints, but inhibits joints). However, we expect this to be a simplification of a more complicated set of interactions that are operating *in vivo.*

We must then introduce a coupling between the dot-forming and stripe-forming subsystems. To match the observations on gene expression, stripes must form in antiphase with the dots, i.e. the coupling should be inhibitory. We achieve this by having the concentration of the dot molecules regulate the reaction parameters of the stripe-forming system, such that dots inhibit stripes (Fig. 1E), taking inspiration from a model of dragonfly vein development (Koch and Meinhardt, 1994). We also consider the more general case where the coupling is bidirectional, such that dots inhibit stripes and stripes inhibit dots (Fig. S1D). However, both models generate qualitatively similar patterns; therefore, we focus here on the simpler case that captures the important phenomenology.

To complete the model, we must specify the geometry on which the coupled dot-stripe system operates: to a first approximation, we assume that joint patterning occurs separately within each digit ray, and use a narrow rod-shaped domain to reflect typical digit shape. To begin with, we do not consider digit growth and instead use a static geometry, in an effort to understand the core self-organizing properties of the dot-stripe model before later incorporating growth dynamics.

Integrating these different components, we then constructed a partial differential equation to describe the dot-stripe mechanism. Simulations revealed that this system was capable of self-organizing into a repeated pattern of dots, interspersed with narrow stripes. Provided a sufficiently narrow digit ray and suitable boundary conditions (see below), these stripes were oriented perpendicular to the hypothetical digit, thus dividing the long rod into a set of repeated units (i.e. phalanges), with the dot molecule expressed central to each phalanx, closely matching the gene expression patterns observed during joint patterning *in vivo* (Fig. 1F). Moreover, the model predicted a subtle modulation of the pattern at the joint edges, with the primary stripes flanked on their edges by short regions of expression (see arrows in Fig. 1F), a pattern that is also seen *in vivo* (Dathe et al., 2009; Huang et al., 2016).

To achieve reliable orientation of joints along the digit, we require that the dot-forming molecule be degraded outside the digit ray (Fig. S1C). Although there is no direct evidence for this in the literature, it is consistent with the observation that ‘Dot’ patterns (e.g. *Ihh*, pSmad) are lowly expressed along the lateral edges of the phalanges, which could be achieved via localized repression by mesenchymal or perichondrial tissue that flanks the digits.

In our simulations, model parameters do not vary along the digit, thus giving rise to phalanges of equal size. This captures the inherently iterative nature of joint patterning, but fails to describe subtle variations in phalangeal size observed *in vivo*, with phalanges becoming progressively smaller along the PD axis across many species (Kavanagh et al., 2013). For now, rather than extending the model to incorporate this variation, we choose to keep the model simple – with a uniform phalanx size within a digit – and focus our attention on qualitative, rather than quantitative, features of the model.

### Combining patterning with limb bud growth

**Fig. 2.**
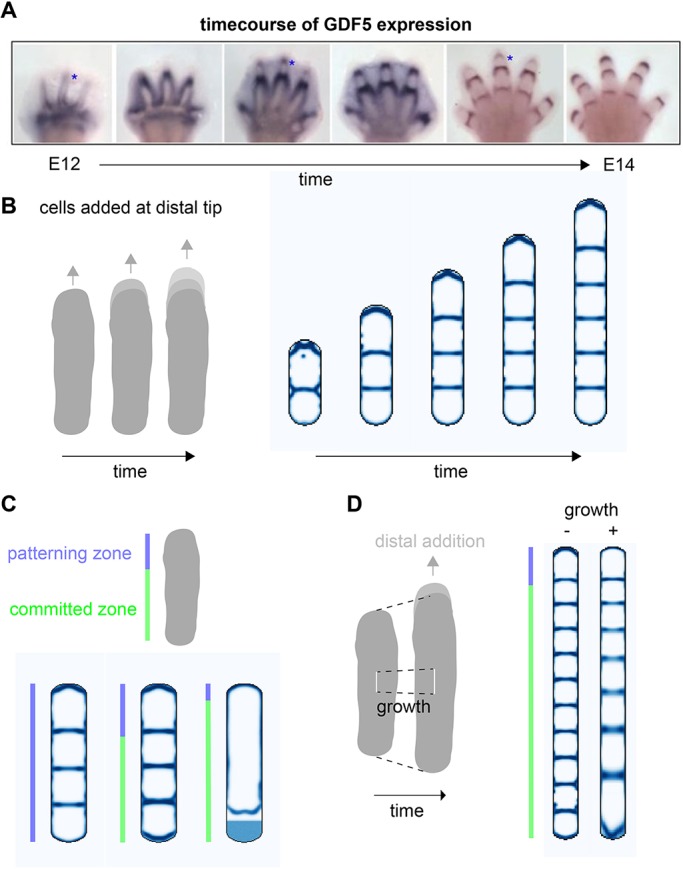
**Digit growth results in sequential joint formation.** (A) Timecourse of GDF5 expression during joint patterning. Reproduced, with permission, from Huang et al. (2016). (B) Simulations incorporating digit growth results in progressive addition of distal joints. (C) A committed zone: final timepoint of simulations that allow patterning only within a certain distance, *L_P_*, to the distal tip; three different values of *L_P_* are shown. (D) Adding uniform growth to the *in silico* digit results in distally decreasing phalanx size.

In our preliminary simulations, joints form simultaneously throughout the domain, whereas *in vivo*, they form sequentially as each digit ray grows outwards (Fig. 2A). To investigate whether this was compatible with the dot-stripe mechanism, we incorporated growth into our model. We achieved this by allowing the domain geometry to vary in time, characterizing a rod-shape digit that progressively grows at its distal tip (Fig. S1E,F). This growth is driven by the incorporation of sub-AER progenitor cells into the distal tip of the digit ray (Huang et al., 2016; Montero et al., 2008; Suzuki et al., 2008). By simulating the dot-stripe mechanism on a growing domain, we saw a change from simultaneous to sequential patterning, with new joints arising at the distal end of the elongating digit ray. A natural consequence of this is that overall digit length is predicted to influence the total number of joints formed, explaining why the hyperphalangeal digits in whale and dolphin flippers are associated with prolonged autopod growth (Richardson and Oelschläger, 2002), and why an extra phalanx is induced when digits are elongated by prolonged *Fgf8* expression in the chick AER (Sanz-Ezquerro and Tickle, 2003).

In these simulations, we observe that patterning is primarily occurring at the distal end of the digit ray, with proximal joints retaining a stable position over time. To investigate this further, we explicitly modelled two zones within the digit – a ‘patterning zone’ at the distal end (akin to the PFR) and a proximal ‘committed zone’ in which cells have irreversibly committed to the joint or phalanx fate. We found that, as long as the patterning zone was at least as large as the typical phalanx size, then joint patterning proceeded normally, suggesting that the dot-stripe mechanism need only operate within the distal digit regions and not the entire digit ray (Fig. 2C).

We further hypothesized that, although cells in the committed region cannot alter their fate, they do continue to proliferate as observed in proximal phalanges (Kavanagh et al., 2013). When we allowed the digit ray to undergo uniform growth in our simulations, while still adding cells at the distal tip, we saw that this generated variations in phalanx size, with the earliest forming proximal phalanges being larger than later forming distal phalanges (Fig. 2D). This provides one hypothesis to explain why there is a progressive decrease in phalangeal proportion from proximal to distal across many species.

Although we have shown that digit elongation drives joint formation distally, the exact location at which a joint first appears depends on model parameters. For example, we observe that if the patterning kinetics are sufficiently fast in comparison with digit growth, then new joints form in the middle of the most distal phalanx, dividing it into two (Fig. S2A). In contrast, for more moderate patterning speeds, new joints are specified at the distal-most edge of the growing digit, and remain stable (Fig. S2A). This second mode is consistent with a detailed study of *Gdf5* expression during mouse joint patterning, highlighting nascent *Gdf5* expression near the distal digit tips (Huang et al., 2016; Ray et al., 2015). We also found that boundary effects could modulate where joints first emerge. In particular, we considered whether high BMP activity around the distal edge of the digit ray could affect joint placement (Suzuki et al., 2008; Montero et al., 2008), and modelled this boundary effect as simultaneously activating dots and inhibiting stripes. When we incorporated this into our model, we observed that this distal modulation could also bias joint formation towards the distal tip, independent of the overall patterning speed. In both cases, the location of the nascent joint is sensitive to model parameters (patterning speed in Fig. S2A; magnitude of boundary effect in Fig. S2B). Further work will be needed to determine the relevant mechanisms at work *in vivo.*

### Prediction of non-intuitive joint orientations

**Fig. 3.**
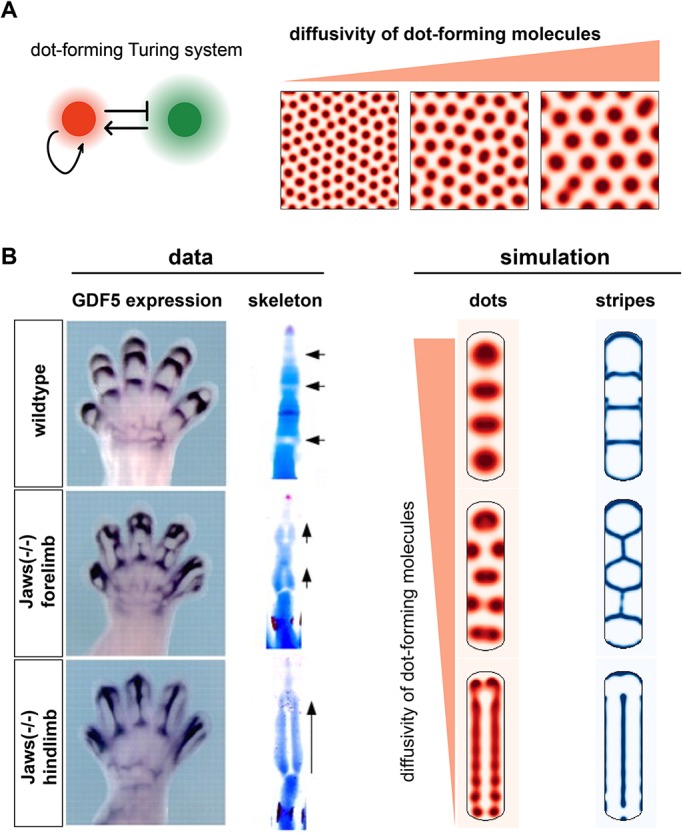
**Prediction of misoriented joints.** (A) Changes to the diffusivity of the dot-forming molecules alters dot spacing. (B) Simulations of the same digit geometry, but altered dot diffusivities, results in aberrant joint morphologies (right) that phenocopy the *Jaws* mutant (left). Arrows indicate joint orientation. Images adapted, with permission, from Sohaskey et al. (2008).

As mentioned above, one way to modulate joint number is to change the overall size of the digit ray. An alternative would be to vary the intrinsic spacing of the dot-forming system, while keeping the final size of the digit constant. In our two-node dot-forming reaction-diffusion model, this spacing is set by the diffusion coefficients of the secreted molecules, as predicted by theory (Hiscock and Megason, 2015a), and confirmed with simulation (Fig. 3A). However, when we decreased the diffusion coefficients of the dot-forming system in our simulations, rather than a simple increase in joint number, we observed non-intuitive results, with some joints forming parallel to the digit, exactly orthogonal to their usual orientation (Fig. 3B). The prediction here is that once the dot spacing is lower than the digit width, then multiple dots can fit side-by-side, thereby forming a joint between them. Moreover, under certain parameter regimes, we predict a single, elongated joint that bisects the digit along its long axis (Fig. 3B). Strikingly, we observe these phenotypes in the *Jaws* mouse mutant (Sohaskey et al., 2008). Disruption of *Jaws* expression results in a generalized loss of extracellular matrix (ECM) integrity, and is associated with defective long-range *Ihh* signalling (*Ihh* is a putative Dot gene, expressed in dots). We hypothesize that *Jaws* regulates dot-spacing by modifying the range of *Ihh* (and perhaps other secreted factors, the transport of which may also be compromised), generating the phenotypes predicted by our model.

### Joint lattices?

**Fig. 4.**
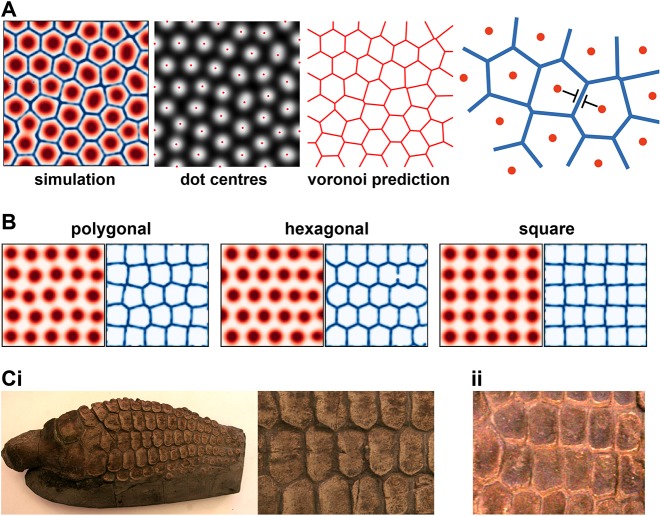
**Joint lattices.** (A) Over a larger domain, the dot-stripe mechanism produces joint lattices where stripes maximize their distance to the nearest dot. (B) For different parameters, joint lattices can be more or less ordered, and polygonal, hexagonal or square. (C) Images of *Ichthyosaur* forefins, revealing lattice-like phalangeal patterns. Samples are from the Sedgwick Museum (University of Cambridge): (i) CAMSM J.47047 *Ichthyosaurus* sp. Lias. Lyme Regis; (ii) CAMSM J.35187 *Ichthyosaurus* sp. Lias. Lyme Regis (collected by Mary Anning).

If we continue to decrease the dot-system diffusion coefficients further, we arrive at a regime in which the intrinsic dot spacing is much smaller than the digit width. Here, the model predicts a connected lattice of joints, interspersed with polygonal-shaped phalanges. The emergence of joint lattices affords a deeper understanding of the dot-stripe mechanism, as illustrated in Fig. 4A, whereby a periodic pattern of dots organizes a lattice of stripes maximally distant from the dot centres (i.e. a voronoi tessellation). In a long, narrow geometry, such as the digit, this predicts a ladder-like joint pattern; whereas in a wide domain, with the same reaction parameters and boundary conditions, a lattice-like pattern forms. Depending on parameters, joint lattices can be ordered or disordered, and polygonal, hexagonal or square (Fig. 4B). Is there evidence for such joint patterns in the tetrapod lineage?

One possibility comes from the group *Ichthyopterygia* (Ichthyosaurs), an extinct group of marine reptiles that returned to the sea during the early Triassic period (Motani, 2005). Ichthyosaur fins have many rows of distal phalanges, sometimes up to 20, e.g. *Ichthyosaurus* (Caldwell, 2002; Motani, 1999, 2005; Sander, 2000). However, unlike the hyperphalangy observed in cetaceans, these distal phalanges are not clearly separated into distinct digits (Motani, 1999); rather, they form a lattice-like structure (Fig. 4C). Based on our mathematical modelling, we hypothesize that these morphologies are in fact joint lattices produced by a conserved dot-stripe mechanism. Under this hypothesis, the ancestral form was a canonical autopod morphology with clear interdigital regions separating the digits, as seen in some of the more basal Ichthyosaur species, e.g. *Utatsusaurus* (Mazin, 1986). Then, some modification resulted in an expansion of the chondrogenic domain and the loss of interdigital regions, such that joint patterning now occurred over a much wider domain – perhaps via an expansion of the mesopodium, as suggested by Woltering and Duboule (2010). This would generate fins with polygonal phalanges separated by joint lattices, as is observed in many Ichthyosaur species, e.g. *Ophthalmosaurus* (Moon and Kirton, 2016).

This hypothesis can explain why, across many Ichthyosaur species with highly variable ‘digit’ numbers (from 2 to 10) and phalangeal rows (up to 20), there is a correlation between the AP spacing of the ‘digits’ and the PD spacing between rows of phalanges (see Sander, 2000). Rather than being distinct processes, we speculate that the AP and PD patterns are formed by the same mechanism, with the characteristic spacing between phalanges (i.e. dots) the same in both directions.

Support for the joint-lattice hypothesis comes from the analysis of disarticulated fins of several species, revealing that phalanges are pitted on all in-plane edges (Caldwell, 1997; Moon and Kirton, 2016; Zammit et al., 2010). Pitted morphology suggests attachment to cartilage and the formation of joints between opposing bony elements, similar to the mesopodium (see below). Being pitted on all sides, rather than just the proximal-distal edges, as in the digits, is consistent with a lattice of interconnecting joints (Caldwell, 1997, 2002).

### Alternative models for joint patterning?

In the previous sections, we have shown that our dot-stripe model can recapitulate joint patterning in wild-type limbs, can explain non-intuitive joint orientations seen in certain mutants, and can generate hypotheses about lattice-like joint morphologies. However, to what extent are these predictions specific to the dot-stripe mechanism? To address this, we considered two alternative models for joint patterning, and asked whether they could also explain the diversity of joint patterns observed *in vivo.*

**Fig. 5.**
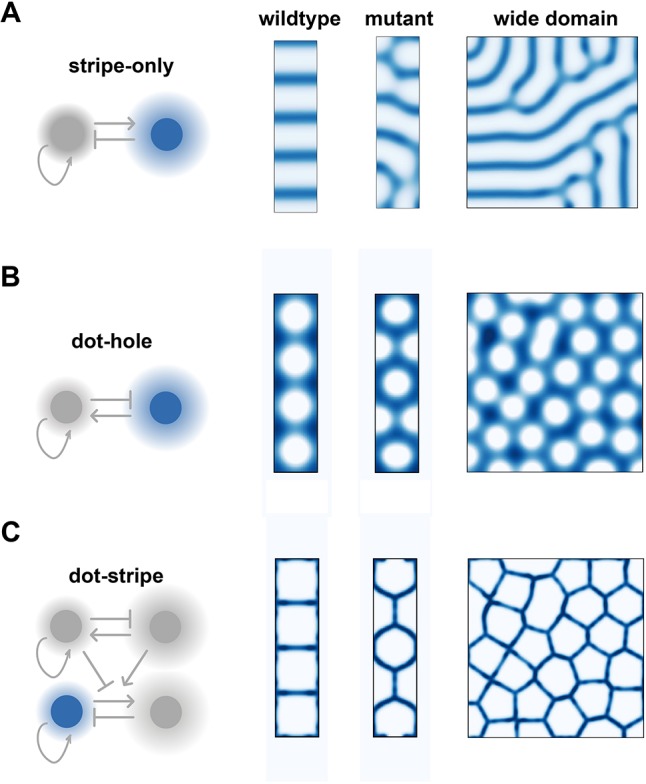
**Alternative models.** (A) A stripe-only model correctly predicts wild-type patterns, but not mutant or lattice-like morphologies. (B) A dot-hole model produces joints of the wrong shape, but connected to one another in the right topology, for both wild-type and lattice-like patterns. (C) A combination of dot- and stripe-forming systems ensures joints form with the correct topology and shape.

First, we modelled joint patterning using a single Turing system that generated stripes. Intuitively a stripe-forming Turing system can naturally explain the periodic placement of interzones along the digit ray in wild-type limbs, which we confirmed with simulation (Fig. 5A). However, this mechanism could not recapitulate joint patterning in *Jaws* mutants nor could it generate joint lattices in broad domains; instead, disorganized striped patterns formed (Fig. 5A).

Next, we considered a model for joint patterning inspired by the dot-forming system in Fig. 1, in which the spaces between the dots, i.e. the holes, specify prospective joints. Unlike the stripe-forming model, the dot-hole model fails to accurately describe patterns in wild-type limbs: regularly spaced interzones appear, but these are curved and broad in contrast to the narrow, straight sites of joint specification observed *in vivo*. However, when considering the mutant phenotype and broader domain, we find that the dot-hole model more closely resembles observed joint patterns than the stripe-only model. In particular, while the precise shapes of the joints are inaccurate, the overall topology of the pattern is correct (Fig. 5B).

Together, these results suggest that, alone, a stripe-only or dot-hole Turing system cannot fully explain the joint phenotypes that are observed *in vivo*, but does provide an insight into why a combination of dot- and stripe-forming systems is required. A dot-forming system specifies the overall topology of the pattern, which then organizes the stripe-forming system to form joints that are locally straight and meet at vertices (Fig. 5C).

### The dot-stripe mechanism as a generic model for joint patterning

Despite the success of the dot-stripe mechanism, the model is highly simplified, composed of only four diffusible factors and the interactions between them. *In vivo* joint patterning involves the cooperation of many diffusible signals, intracellular signal transduction cascades and transcription factor networks. Moreover, behaviours at the level of cells (e.g. proliferation, migration) and tissues (e.g. mechanical compression; Singh et al., 2018) are also likely to be important, as the interzone is characterized by a region of increased cell density in addition to its molecular signatures. How then do we reconcile the complexity of joint patterning *in vivo* with our simplified model?

We take inspiration here from work on the classic Turing mechanism (Turing, 1952). In its original conception, the Turing model consisted of two interacting and diffusing molecules, capable of self-organizing into dots or stripes. In the decades since, many iterations of this simple model have been proposed, including more complicated molecular circuits in the limb (Raspopovic et al., 2014), models that include cell migration and chemotaxis in epidermal appendages (Painter et al., 2018), and mechanical instabilities in the intestine (Shyer et al., 2013). In each case, although the details are different, there is a common logic that is sufficient to generate dots or stripes: local activation and long-range inhibition. Theoretical work has formally shown that these diverse models fall into the same broad category of mechanisms, and can be described by a generic model of periodic patterning: the Swift-Hohenberg equation (Cross and Hohenberg, 1993; Hiscock and Megason, 2015a).

**Fig. 6.**
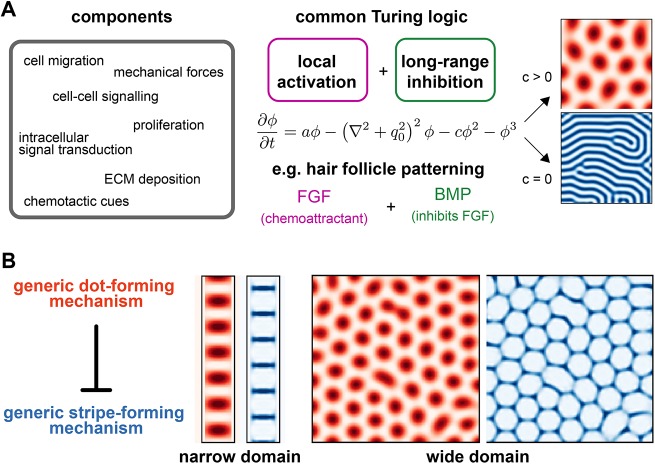
**A generic dot-stripe model.** (A) Many different biological processes (left) can form Turing patterns provided a core Turing logic is satisfied (middle). This can be modelled by the Swift-Hohenberg equation, which can generate either stripes or dots (right). (B) A generic dot-stripe model can recapitulate joint patterns along a narrow digit-shaped domain and joint lattices over a wider domain.

We aimed to construct a generic model of joint patterning that would capture the core logic of our previous model, but would generalize to more complicated and realistic biological mechanisms. We used two versions of the Swift-Hohenberg equation: one as a generic way to generate dots and one as a generic way to generate stripes (Fig. 6A). We then coupled these two systems together by having the parameters of the stripe system be modulated by the dot system, so that stripes would only form in the absence of a dot. We found that two parameters must vary: (1) the Turing instability parameter *a*, which controls whether or not stripes will form; and (2) the overall bias parameter *h*, which governs the orientation of the stripes (Fig. S4) (Hiscock and Megason, 2015b).

Simulations of this generic dot-stripe model captured the same phenomenology of joint patterning as before, with narrow geometries resulting in iterative joints reminiscent of the digits and wider domains giving rise to joint lattices (Fig. 6B). This suggests that the dot-stripe mechanism represents a rather generic mechanism to make joint patterns, and may rely on a range of different molecular, cellular and mechanical interactions.

## DISCUSSION

In this work, we have developed a mathematical model of joint patterning that can explain the location, number and orientation of joints within the developing digits of various species and mutants. Central to our model is the proposal that the joint-forming mechanism is inherently iterative, such that a repeated set of joints self-organizes from an initially unpatterned state. In our model, a dot-forming Turing system is responsible for symmetry breaking, forming a series of evenly spaced dots along the digit ray. These then instruct a set of Turing-stripes – which will ultimately give rise to the mature joints – to form between the dots. The intrinsic periodicity of the joint programme allows for large variations in phalangeal number (up to 17 in a single pilot whale digit; Cooper et al., 2007), by changing either the dot-spacing or the distal extension of the digit.

As previously discussed, in its simplest form the dot-stripe mechanism predicts phalanges that are equal in size along the digit, something that is rarely observed *in vivo.* In many species, phalangeal proportions decrease gradually from proximal to distal (Kavanagh et al., 2013). This general trend is marked by exceptions in which phalangeal size is highly non-uniform and often associated with specialized digit function (e.g. perching). Unequal phalangeal proportions could be explained by the dot-stripe model provided the parameters that control joint spacing vary along a digit, presumably under the influence of PD patterning cues (e.g. *Fgf* and 5′Hox genes). Growth may also play a role, given that the proximal phalanges are formed first, and appear to increase their size at the same time as distal phalanges are being specified (see Fig. 2A); we explore this proposal with simulations in Fig. 2D. Further work is needed to better understand which mechanisms allow joint spacing to vary within a digit.

**Fig. 7.**
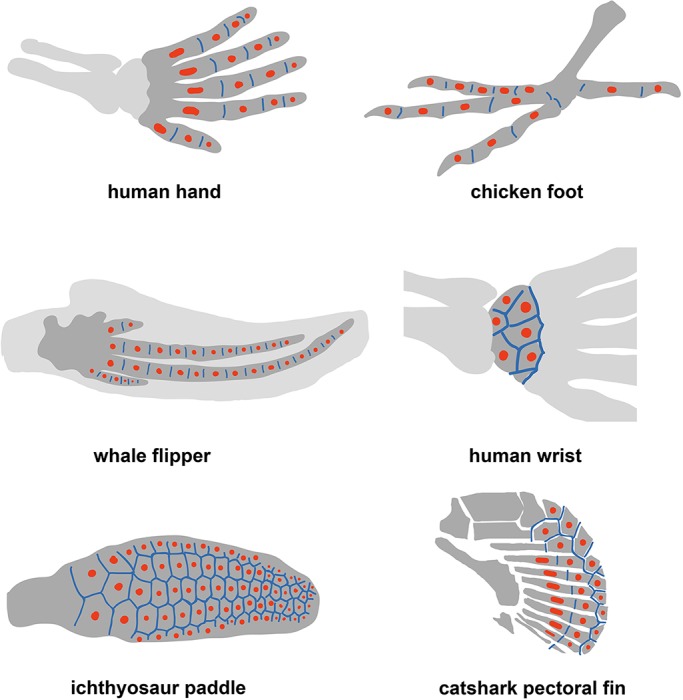
**An ancestral dot-stripe mechanism may explain diversity of skeletal morphology in limbs and fins.** Schematics of limbs and fins for different species are drawn. Putative dot (red) and stripe (blue) expression patterns are indicated.

Similarly, modulation of joint spacing along the AP axis of the limb bud may occur (downstream of Shh/Bmp/5′Hox gene gradients), and this offers a reinterpretation of the concept of digit identity in the chick. In the French Flag model, a gradient of SHH activity specifies different types of digit at different positions across the AP axis of the limb, characterized by having different numbers of phalanges (see Fig. 7). Within the dot-stripe framework, discrete digit morphologies can be produced by varying the intrinsic joint spacing along the AP axis of the limb.

Our model also provides insight into the regulation of joint orientation. For a rod-shaped digit, joints are predicted to form perpendicular to the digit, forming hinges along its length. However, our simulations reveal that variations to digit geometry or model parameters can substantially alter joint orientations. Longitudinal joints are predicted and observed in the *Jaws* mouse mutant. Similar examples of misoriented joints have been reported in whale flippers with aberrant digit-interdigit patterning (Cooper and Dawson, 2009), and may also explain the split phalanx phenotype seen in *Gli3^−/−^* mutants (Huang et al., 2016). Furthermore, in the Ichthyosaur fin, we speculate that broadening of the chondrogenic region results in a polygonal arrangement of phalanges connected by a lattice of joints with variable orientations, forming a structure of increased mechanical rigidity.

These theoretical predictions also provide a hypothesis for how joints form in the mesopod. Carpals and tarsals, the bones of the wrist and ankle, respectively, are formed by the splitting of large chondrogenic domains into individual elements (Akiyama et al., 2002; Shubin and Alberch, 1986). These elements are roughly polygonal in shape when formed, and are flanked on all sides by *Gdf5*-expressing synovial joints (Chen et al., 2016), resembling the polygonal joint lattices predicted by the self-organizing dot-stripe model (Fig. 7). Therefore, we propose that the same type of mechanism is patterning joints in both the autopod (digits) and the mesopod (carpals/tarsals), but with different parameters and geometry. In support of this hypothesis, an increased number of carpals/tarsals is observed when the mesopodial field is enlarged (Muragaki et al., 1996; Sheth et al., 2007; Whited et al., 2013; Woltering and Duboule, 2010), as expected for the dot-stripe mechanism in which the characteristic element number is not hardcoded but controlled by the intrinsic dot spacing. Further characterization of the molecular regulation and histogenesis of wrist development will be necessary to test this hypothesis.

Despite the success of the dot-stripe mechanism in capturing the phenomenology of joint patterning, its molecular underpinnings remain unspecified. In this regard, the model should not be viewed as a description of a known molecular mechanism, but rather as a guide that tells us what form of molecular mechanism we should expect. For example, the expression of conflicting signals from within the developing joint – *Wnt* ligands that promote joint formation and *Gdf5* that inhibit it – is compatible with the Turing-type logic required to form stripes, as suggested by our model. Moreover, we predict the existence of a dot-forming mechanism; this likely involves BMP signalling within the phalanx centre, and perhaps also *Ihh*, although the latter is dispensable for patterns to form (Hilton et al., 2005; Koyama et al., 2007). However, for both dot and stripe systems, a complete molecular picture is lacking. Providing a detailed mechanistic description of the dot-stripe mechanism – at the level of molecular networks and cellular behaviours – should form the focus of future work, and can be guided by the model presented here. Transcriptomic approaches [e.g. single cell RNA sequencing (Feregrino et al., 2019) and spatial transcriptomics (Rodriques et al., 2019)] may prove particularly effective, allowing for a comprehensive enumeration of the signalling pathways involved. It will also be important to compare the model with the process of digit-interdigit patterning, which relies on many of the same signalling pathways (Hiscock et al., 2017; Raspopovic et al., 2014), and to consider the molecular regulation and cellular processes driving digit growth.

Finally, our model can sharpen questions regarding the fin-to-limb transition and the emergence of joint patterning during evolution. Recent evidence suggests that synovial joints – thought to be characteristic of the tetrapod limb – are also found in the pectoral fins of some ray finned fishes (Askary et al., 2016). Moreover, analysis of HoxA/D expression in teleosts and chondricythans suggests that the tetrapod autopod/mesopod shows deep homology with the distal radials of the paired-fin endoskeleton of other gnathostomes, hinting that there may be conserved patterning modules (Freitas et al., 2007; Tulenko et al., 2016). Indeed, Onimaru and colleagues propose that the distal radials of chondricythan pectoral fins are patterned by the same mechanism that controls digit-interdigit patterning in tetrapods (Onimaru et al., 2016). We speculate that there could also be a conserved joint-patterning module, in which a common dot-stripe mechanism operates with different parameters and geometries to generate distinct fin/limb morphologies. Consistent with this hypothesis, chondricthyan pectoral fins contain long, rod-shaped elements that are jointed along their length, similar to the tetrapod digits. However, they also contain elements that are polygonal in shape and articulate to several other elements, similar to the tetrapod wrist (Fig. 7); e.g. the polygonal plates of the catshark (Freitas et al., 2007) or the metapterygium of the skate (Dahn et al., 2007). Future investigation of joint patterning in these species will be necessary to test to what extent the dot-stripe mechanism has been reused throughout evolution to generate novel skeletal morphologies and functions.

## MATERIALS AND METHODS

### Formalizing the dot-stripe mechanism

To model the spatiotemporal dynamics of the dot-stripe mechanism, we formulated a set of partial differential equations (PDEs) that capture the putative interactions inferred from *in vivo* expression data and perturbations (Fig. 1). For the dot-forming system, we chose a canonical activator-substrate Turing system (Gierer and Meinhardt, 1972). Specifically, we modelled two molecular species: *A*, which form dots; and *S*, which form holes (inverted dots). The mathematical description of the model (outlined in Fig. 1C) entails the following behaviours: (1) *A* represses *S*, controlled by parameter *k_S_*; (2) *S* activates *A*, controlled by parameter *k_A_*; (3) *A* and *S* diffuse with coefficients *D_A_* and *D_S_*, respectively; (4) A is degraded at rate *k_A_*; and (5) both display generic, concentration-independent activation (*h_A_* and *h_S_*):(Eqn 1A)
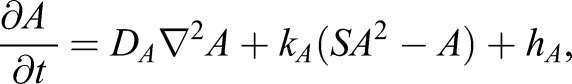
(Eqn 1B)
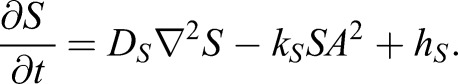


For the stripe model, we used a modified version of the classic activator-inhibitor model (Gierer and Meinhardt, 1972). Here, an activator molecule, *B*, and an inhibitor molecule, *I*, are both expressed in striped patterns governed by the following behaviours (Fig. 1D): (1) *B* undergoes self-activation, controlled by parameter *k_B_*; (2) this self-activation is repressed by *I*; and (3) is self-limiting at high *B*, controlled by *κ*_*B*_; (4) *B* activates *I* (*k_I_*); (5) both *B* and *I* undergo diffusion (*D_B_*, *D_I_*); and (6) both *B* and *I* are degraded (*k_B_^*0*^*, *k_I_*). These dynamics are described by:(Eqn 2A)
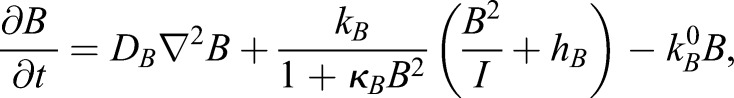
(Eqn 2B)
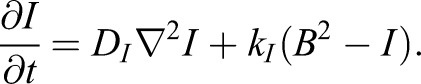


To couple the two systems such that stripes form between dots, we take inspiration from a model of dragonfly vein development (Koch and Meinhardt, 1994), allowing *S* to activate *B* and *A* to increase the self-activation threshold of *B* (i.e. a repressive effect). In this, *B* stripes are favoured at distances maximal from *A* dots, and are modelled by having:(Eqn 3A)
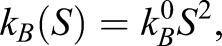
(Eqn 3B)
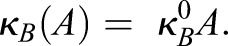


This gives a total set of equations:(Eqn 4A)
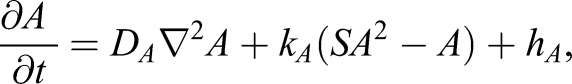
(Eqn 4B)
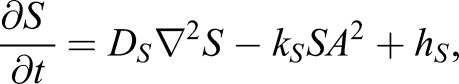
(Eqn 4C)
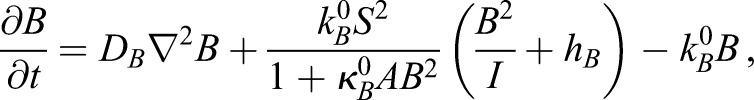
(Eqn 4D)
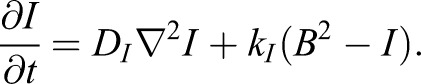


This system, given appropriate parameters, will self-organize into a set of evenly spaced dots interspersed by narrow stripes (Fig. 1F). We chose the homogeneous steady-state solutions as initial conditions.

The final component of simulating this PDE was to specify the domain and boundary conditions; simulations revealed that this choice affected the pattern of stripes formed. Here, we assumed that the Turing molecules were produced only within the developing digit ray (as motivated by expression of the genes in Table S1). However, we assumed that, once produced, these molecules would diffuse out of the digit ray and into neighbouring mesenchymal regions, where they would be subject to degradation. We found that a single degradation rate – *k*_*deg*_, specifying the degradation of *A* outside of the digit – was sufficient to recapitulate wild-type joint patterns, with other degradation terms having minimal qualitative effects on the patterning, and were thus ignored for simplicity.

We formalize this with a separate set of PDEs governing the dynamics outside of the prescribed digit geometry:(Eqn 5A)
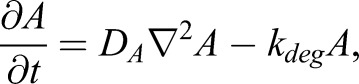
(Eqn 5B)
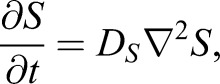
(Eqn 5C)
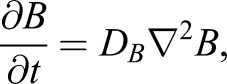
(Eqn 5D)
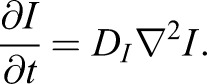


We found that a certain range of *k*_*deg*_ resulted in dots central to the digit ray and stripes that divided the narrow digit (Fig. S1C). For a full summary of all variables and parameters used, see Table S2.

### Digit geometry and growth

In this framework, digit shape is specified by a binary matrix, *Ω*, which is zero everywhere outside the domain, one inside the domain and determines whether Eqns 4 (*Ω*=1) or 5 (*Ω*=0) are employed. We approximate digit shape as a rectangle of length *L* and width *W* that is rounded on both ends by a half-ellipse of length *ε* (and width *W*).

Growth is incorporated by allowing *Ω* to change over time. Here, we assume that growth is entirely along the digit length, and allow the rectangle length to vary linearly in time from *L*_0_ to its final size *L*. We vary the relative speeds of patterning and growth by varying the overall time *T* of the simulation, while keeping *L*, *L*_0_ fixed. We choose the initial conditions to be homogeneous steady states of Eqn 4 combined with stochastic noise, and allow a short time *T*_*i*_ for the pattern to settle before allowing growth to start.

Apart from Fig. 2D, we model elongation of digits at their distal tip only, with molecular concentrations being unaffected by growth except at the very tip. We assume that as new material enters the patterning domain at the tip, it retains its prior state in the first time-step upon entering the digit ray. We note that alternative ways to specify the initial conditions of the new material (e.g. by duplicating distal elements) had little effect on the patterning dynamics.

To model the effect of a commitment zone (Fig. 2C), we fixed all molecular concentrations (*A*, *S*, *B* and *I*) once they were a certain distance from the end of the digit ray (*L*_*P*_). Although these variables are held constant, they still affect patterning via diffusion into the distal regions.

We modelled the boundary effect of the digital crescent as a narrow, curved domain at the tip of the growing digit with its own set of PDEs (Fig. S2B), which model localized activation of *A* with rate *h_A_^DC^* and a localized repression of *B* with rate *h_B_^DC^*:(Eqn 6A)
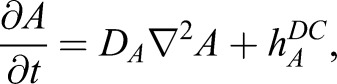
(Eqn 6B)
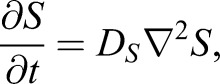
(Eqn 6C)
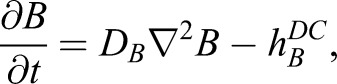
(Eqn 6D)
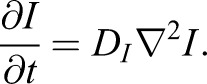


To model uniform digit growth, we computationally ‘stretched’ the digit a small amount along its length at regular time intervals. In Fig. 2D, for every increment in digit length, we alternated between stretching the digit (uniform growth) and extending the digit at its tip (distal elongation); the relative frequencies of these two modes can modulate the ratio of distal versus uniform growth.

### Simulation methods

We solved Eqns 4A-D and 5A-D on a large rectangular domain, discretized into square bins. Different geometries are specified within this larger domain by a binary matrix *Ω*: for *Ω*=1, Eqns 4A-D are used; for *Ω*=0, Eqns 5A-D are used. The system of PDEs is then solved using an operator splitting method: at each time-step, *δt*, diffusion is simulated by combining the backward Euler method with the discrete cosine transform, which models reflective boundary conditions at the edge of the domain. Then, the reaction terms are calculated by applying the forward Euler method. The simulation is iterated until a final time *T* is reached.

### Simulation parameters

Parameters were initially chosen such that the dot-stripe system could spontaneously break symmetry; we took the parameters from Koch and Meinhardt (1994) as a starting point. To generate different patterns, we varied parameters manually, targeting parameters that we expected to have the desired effect. To change the pattern wavelength, we varied diffusion constants, but kept reaction parameters the same: for the dot-forming system, we varied the magnitude of *D*_*A*_, *D*_*S*_ while keeping their ratio constant; for the stripe-forming system, we varied the magnitude of *D*_*B*_, *D*_*I*_ while keeping their ratio constant. To change the effect of the boundary condition, we varied the magnitude of *k*_*deg*_. We altered digit geometry by varying *W*, *L*_0_, *L*, *ε*. We altered the speed of patterning compared with growth by keeping geometry parameters fixed and varying *T*. For full details of parameters used, see Table S3.

### Analysis of the dot-stripe model

#### Voronoi tessellations emerge from the dot-stripe mechanism

Based on the geometry of the joint lattices (Fig. 4B), we hypothesized that the dot stripe could give rise to Voronoi tessellations. To test this, we found the local maxima in the dot-pattern and then computed the predicted Voronoi tessellation from these points; we found good agreement with the simulated pattern (Fig. S3C).

#### Analysis of the interaction between dot- and stripe-forming systems

We then interrogated the model further by directly controlling the (*A*, *S*) system. First, we set (*A*, *S*) to uniform values across the entire domain, and asked how the (*B*, *I*) system responded. We did this for a range of (*A*, *S*) values that corresponded to being close to or far from the dot centres (defined above), respectively. We see that when the (*A*, *S*) values are chosen to mimic being close to the dot centre (*A*=high, *S*=low), then the (*B*, *I*) system failed to form robust stripes. As the effective distance to dot centre increased (*A*=low, *S*=high), we see that the (*B*, *I*) system begins to robustly self-organize stripes, with an increasing amplitude (Fig. S3A). This confirms our interpretation that dots are inhibiting stripes such that stripe self-organization occurs preferentially in regions far from dot centres.

We then controlled the spatial variation of (*A*, *S*) values. We simulated a one-dimensional gradient, going from (*A*=high, *S*=low) to (*A*=low, *S*=high), i.e. moving away from the dot centre. We saw, as above, that stripes formed far from the dot centre. Moreover, the stripes had a defined orientation: they were perpendicular to the direction of change (Fig. S3B). This makes sense – in the full dot-stripe model, not only must stripes organize between dots, but their orientation must be perpendicular to dot spacing such that they bisect the dots.

### Alternative models

In Fig. 5, we consider alternative models for joint patterning. For the stripe-only model, we use Eqns 2A,B, but now with fixed values of *κ*_*B*_ and *k*_*B*_, and identify *I* as the joint marker. We choose reflective boundary conditions that result in alignment of stripes perpendicular to the digit. To generate disorganized stripes, we either vary the diffusion constants (*D_B_*, *D_I_*) that control stripe spacing or increase the width of the domain.

For the dot-hole model, we use Eqns 1A,B within the digit and 5A,B outside the domain, and identify *S* as the joint marker. We use the same (static) domain geometries as in Fig. 5A to facilitate direct comparison, and modify diffusion constants (*D_S_*, *D_A_*) or domain width to alter joint patterns.

For the dot-stripe model (Fig. 5C), we use Eqns 4 and 5, and identify *B* as the joint marker. Similar to Fig. 5B, we change either *D_S_*, *D_A_* or domain width to model variations in joint patterns.

### A generic dot-stripe mechanism

The above analysis identified two key features of the dot-stripe mechanism. Stripes form: (1) away from dots; and (2) perpendicular to dot spacing. We aimed to capture these dynamics using a generic, one-variable model for each of the Turing systems.

For the dot system, we use the generalized Swift-Hohenberg equation (Burke and Knobloch, 2006), defined by the single variable, [DOT]:(Eqn 7)
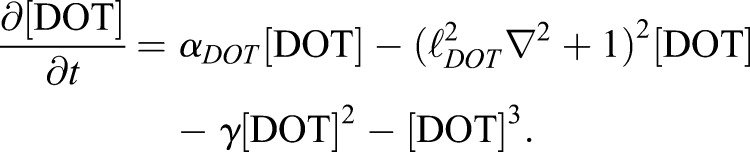


Here, *α*_*DOT*_>0 guarantees that dots will form, ℓ_*DOT*_ sets the approximate dot spacing, and *γ* ensures the resultant pattern is dots, not stripes.

For the stripe system, defined by variable [STRIPE], we use the Swift-Hohenberg equation, with an additional bias term *h*_*STRIPE*_ to promote stripe formation, and relative timescale parameter *τ* to modulate the relative speed of the stripe-equation relative to the dot-equation:(Eqn 8)
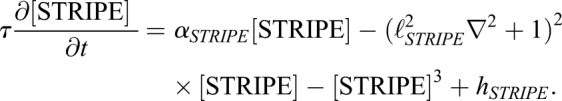


We then coupled the two systems. First, we tried the most straightforward repression of [STRIPE] by [DOT], i.e. *h*_*STRIPE*_([DOT]) as a decreasing function of [DOT]. This generated a hole-like pattern (Fig. S4A):(Eqn 9)
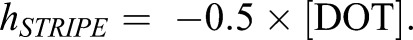


Next, we tried varying the Turing instability parameter, such that *α*_*STRIPE*_([DOT]) was a decreasing function of [DOT]:(Eqn 10)
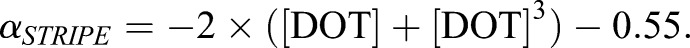


This mirrored the results from Fig. S4B, allowing stripes to self-organize between dots. However, this coupling led to stripes with the wrong orientation (parallel to the spacing of dots, rather than perpendicular), as predicted (Hiscock and Megason, 2015b). Finally, we combined these two couplings to generate stripes: (1) between dots; and (2) oriented parallel to dot spacing. By having *h*_*STRIPE*_([DOT]) and *α*_*STRIPE*_([DOT]) both as decreasing functions of [DOT], we could recapitulate the phenomenology of the four-gene dot-stripe model (Fig. S4C). For the simulations on the narrow domain, we specified an additional degradation of [DOT] outside the domain, ensuring dot centrality similar to Fig. S1C. We note that the stripes formed are not as straight as in the four-gene model, but are significantly straighter than a dot-hole model (Fig. 5B). The decrease in stripe straightness is not unexpected, as the full dot-stripe model is highly nonlinear (compare with the *B*^2^/*I* term), whereas the Swift-Hohenberg equation has only cubic nonlinearities.

### Swift-Hohenberg simulation parameters

The generic dot-stripe mechanism was simulated with the following constant parameters:
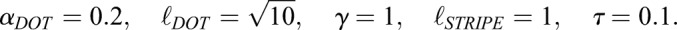


For the case of uncoupled dot and stripe systems (Fig. 6A), we used:
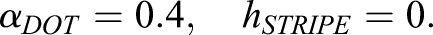


In Fig. S4A, we set either *α*_*STRIPE*_ = 0; in Fig. S4B, we set *h*_*STRIPE*_ = 0.

### Code

Simulation scripts are written in MATLAB and are available at https://github.com/jakesorel/dot_stripe.

## Supplementary Material

Supplementary information

Reviewer comments
