# Supplementary material for: A dot-stripe Turing model of joint patterning in the tetrapod limb
Source: Development. 2020 Apr 12;147(8):dev183699. doi: 10.1242/dev.183699 (PMC7174842; doi:10.1242/dev.183699)
Supplement: Supplementary information [file develop-147-183699-s1.pdf]

Table S1: categories of gene expression pattern within the developing digit.

Type	Gene	Early expression	Perturbation
Stripes	Gdf5	(Merino et al., 1999; Storm & Kingsley, 1999; Gao et al., 2009; Ray et al., 2015; Huang et al., 2016)	• Gdf5 beads embedded adjacent to nascent joints inhibits joint formation in chick (Merino et al., 1999) and mouse (Storm and Kingsley, 1999)<br>• Gdf5(-/-) mice show ectopic joint initiation (Storm and Kingsley, 1999)
	Wnt9a	(Kan & Tabin, 2013; Sohaskey et al., 2008)	• Retroviral mis-expression of Wnt9a in chick induces ectopic joint formation and downregulates chondrogenesis (Hartmann & Tabin, 2001)
	Wnt16	(Kan & Tabin, 2013)	• WNT/ß-catenin signalling is necessary and sufficient to form joints (Guo et al., 2004)
	PthrP	(Gao et al., 2009)	
	Chordin	(Kan & Tabin, 2013)	
	cJun	(Kan & Tabin, 2013)	• Cre mediated deletion of cJun from early mouse limb mesenchyme disrupts interzone formation and Wnt9a/Wnt16 interzone expression (Kan and Tabin, 2013)
Dots	pSMAD1/5/8	(Huang et al., 2016)	• Activation of the BMP pathway inhibits joint formation (Brunet et al., 1998; Zou et al., 1997)<br>• Inhibition of the BMP pathway expands joint progenitors (Yi et al. 2008)<br>• In chick, interdigital sources of BMP, both endogeneously present and exogeneously applied, affect joint patterning in nearby digits (Dahn and Fallon, 2000; Suzuki et al., 2008)
	Ihh	(Gao et al., 2009)	• Loss of joints in Ihh(-/-) mice (Hilton et al., 2005)<br>• Partial rescue of joints in Ihh(-/-); Gli3 (-/-) double mutant mice<br>• Mouse Ihh E95K mutation, reducing capacity and range of hedgehog signalling, leads to loss of middle phalanx from digit V, and spreading of Gdf5 expression (Gao et al., 2009)
	Ppr	(Gao et al., 2009)	
Holes	Hip1	(Gao et al., 2009)	
	Gli1	(Gao et al., 2009)	

Development • Supplementary information

Table S2: Summary of model variables and parameters

Category	Type	Description
Dot system	A	Concentration of dot molecule
	S	Concentration of hole molecule
	D_A	Diffusivity of *A*
	D_S	Diffusivity of *S*
	k_A	Controls degradation and production of *A*
	k_S	Controls degradation and production of *S*
	h_A	Concentration-independent production of *A*
	h_S	Concentration-independent production of *S*
	k_{deg}	Degradation rate of *A* outside domain
Stripe system	B	Concentration of activating stripe molecule
	I	Concentration of inhibitory stripe molecule
	D_B	Diffusivity of *B*
	D_I	Diffusivity of *I*
	h_B	Controls production of *B* that is independent of *I*
	k_B^0	Controls degradation and production of *B*
	k_I	Controls degradation and production of *I*
	κ_B^0	Controls the inhibitory effect of *A* and *B* on the production of *B*
Geometry	L_0	Initial digit length
	L	Final digit length
	W	Digit width
	ϵ	Semi-minor axis (half-width) of ellipses at digit ends
	T	Total simulation time
	T_i	Time to allow patterns to settle without growth
	L_P	Length of patterning region

Development • Supplementary information

Table S3: Simulation parameters

Unless otherwise stated, we kept the following parameters constant across all simulations:

$k_A = 0.0025, k_S = 0.003, k_B^0 = 0.01875, k_I = 0.0375\ h_A = 0.00025, h_S = 0.003, h_B = 0.00187, \kappa_B^0 = 0.2, k_{deg} = 1 \times 10^{-5}, \epsilon = 3, T_i = 5e3, \delta t = 20$

Figure	D_A	D_S	D_B	D_I	L_0	L	W	T	other
**1C,D**	0.008	0.16	0.006	0.12	128	128	128	12e4	$k_B = k_B^0 S_0^2, \kappa_B = \kappa_B^0 A_0, \epsilon = 0, T_i = 0$
**1F**	0.008	0.16	0.006	0.12	128	128	20	6e4	-
**2B**	0.0007	0.0135	0.0004	0.0076	5	36	6	12e4	-
**2C**	0.002	0.039	0.0011	0.0219	5	40	10	12e4	$L_P = \infty, 12{,}2$
**2D**	0.0018	0.036	0.001	0.0203	5	115	10	24e4	$T_i = 2e4$
**3A (left)**	0.004	0.08	-	-	128	128	128	12e4	-
**3A (middle)**	0.008	0.16	-	-	128	128	128	12e4	-
**3A(right)**	0.016	0.32	-	-	128	128	128	12e4	-
**3B (upper)**	0.0015	0.03	0.0008	0.0169	10	34	10	6e4	$T_i = 1e3$
**3B (middle)**	0.001	0.02	0.0008	0.0169	10	34	10	6e4	$T_i = 1e3$
**3B (lower)**	0.0004	0.0075	0.0008	0.0169	10	34	10	6e4	$T_i = 1e3, k_{deg} = 6 \times 10^{-5}$
**4B (left)**	0.002	0.039	0.0015	0.0292	50	50	50	20e4	$T_i = 2e4, \epsilon = 3$
**4B (middle)**	0.002	0.039	0.0015	0.0292	10	50	50	20e4	$T_i = 2e4, \epsilon = 3, k_{deg} = 1 \times 10^{-6}$
**4B (right)**	0.002	0.039	0.0015	0.0292	10	50	50	20e4	$T_i = 2e4, \epsilon = 3$
**5A (left)**	-	-	0.00112	0.2250	40	40	10	6e4	$k_B = 0.0128, \kappa_B = 0.22, \epsilon = 0$
**5A (middle)**	-	-	0.0079	0.1575	40	40	10	6e4	$k_B = 0.0128, \kappa_B = 0.22, \epsilon = 0$
**5A (right)**	-	-	0.0056	0.1125	40	40	40	6e4	$k_B = 0.0128, \kappa_B = 0.22, \epsilon = 0$
**5B (left)**	0.0018	0.036	-	-	40	40	10	12e4	$T_i = 1e3, \epsilon = 0$
**5B (middle)**	0.0012	0.024	-	-	40	40	10	12e4	$T_i = 1e3, \epsilon = 0$
**5B (right)**	0.0027	0.054	-	-	40	40	40	12e4	$T_i = 1e3, \epsilon = 0$
**5C (left)**	0.0018	0.036	0.001	0.0203	40	40	10	12e4	$T_i = 1e3, \epsilon = 0$
**5C (middle)**	0.0012	0.024	0.001	0.0203	40	40	10	12e4	$T_i = 1e3, \epsilon = 0$
**5C (right)**	0.0027	0.054	0.0015	0.0304	40	40	40	12e4	$T_i = 1e3, \epsilon = 0$
**S1C**	0.0008	0.16	0.006	0.12	128	128	20	6e4	$k_{deg} = 0, 1 \times 10^{-5}, 1 \times 10^{-4}$
**S1D**	0.0008	0.16	0.006	0.12	128	128	20	6e4	$SA^2 \rightarrow SA^2(1 + 0.1B^2)^{-1}$ in Equation 1a,b; 4a,b
**S2A**	0.002	0.039	0.0011	0.0219	5	50	10	->	$T = 12e4, 3.5e4, 1e4$ from left to right
**S2B**	0.0024	0.048	0.0018	0.036	5	50	10	20e4	$h_A^{DC} = 0, 0.0004, 0.0006,\ h_B^{DC} = 0.005$

Development • Supplementary information

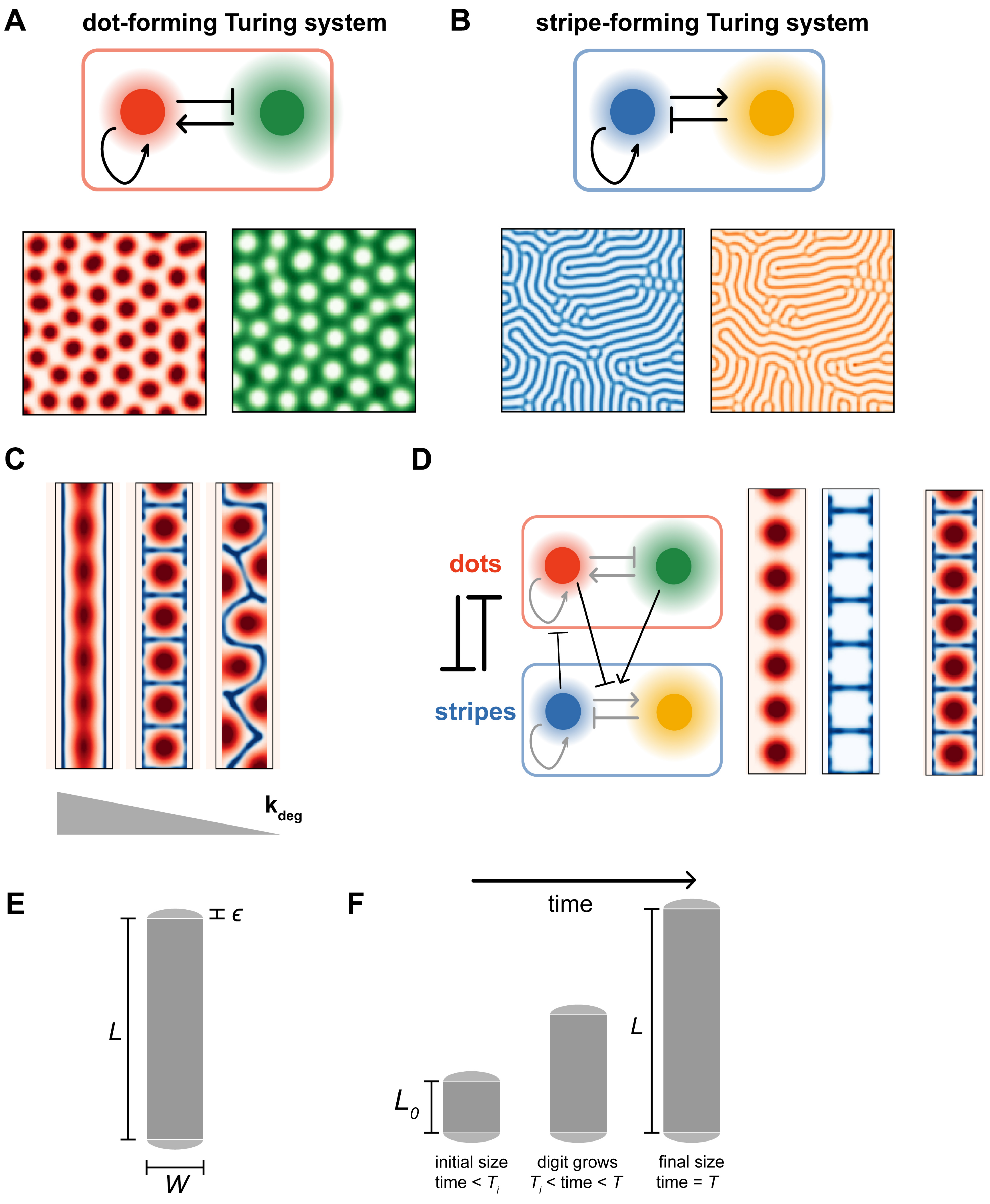

Development • Supplementary information

Figure S1

(A) In the dot-forming system, dots (red) form in antiphase with holes (green).

(B) In the stripe-forming system, stripes (blue) form in-phase with other stripes (orange).

(C) Varying the degradation rate of *A* outside the domain (k_{deg}) changes joint orientation. An intermediate value is required to get stereotypical joint morphology.

(D) A more general model, involving mutual repression between the dot- and stripe-systems, generates patterns that are qualitatively similar to the simpler model in Fig. 1.

(E,F) Schematic of parameters describing digit geometry and growth.

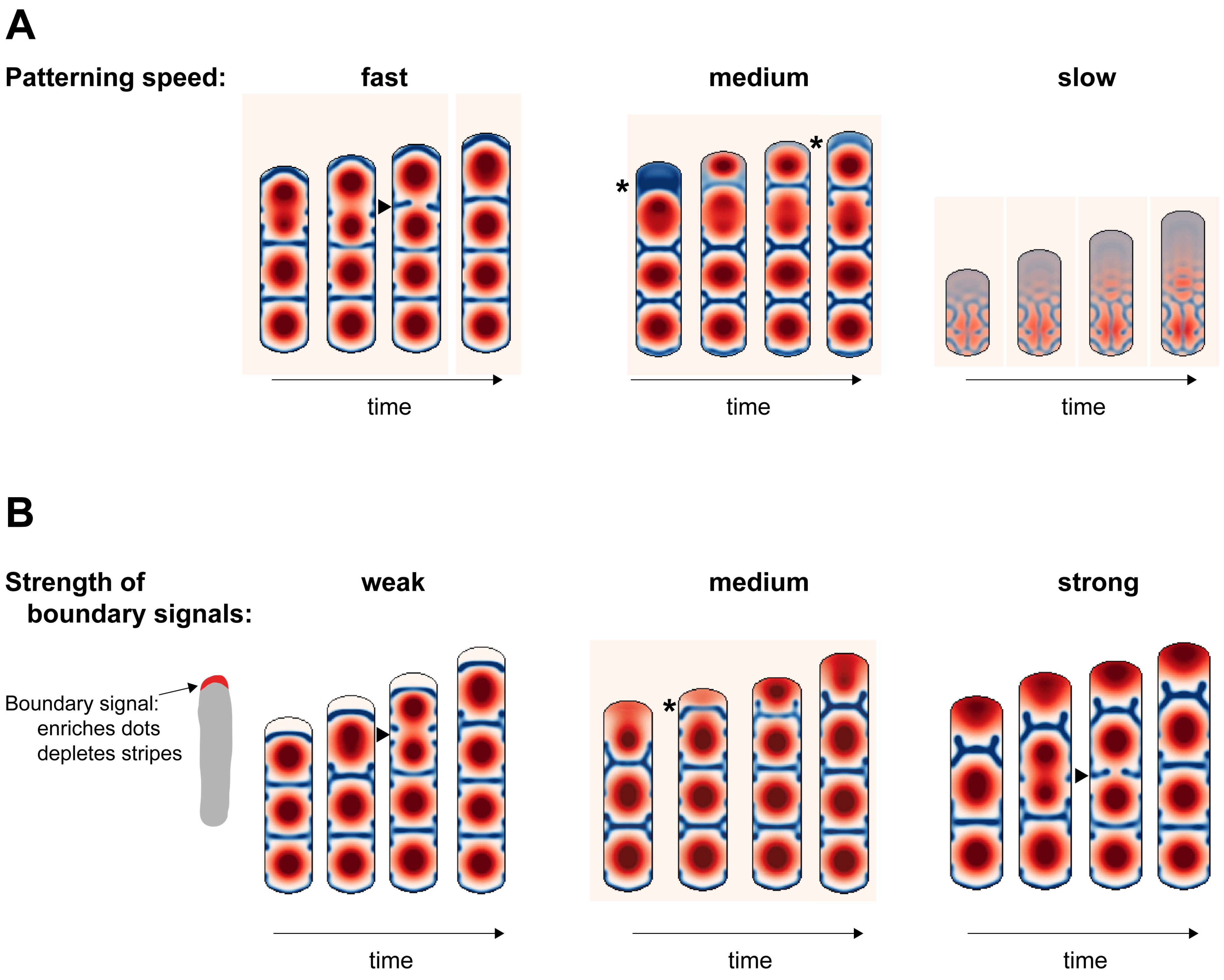

Figure S2

(A) Changing the speed of patterning modulates the precise location of newly forming joints. Left: fast patterning results in joints that divide the distal phalanx (arrowhead). Middle: slower patterning results in joint specification at the growing tip (asterisk). Right: if patterning is too slow, the system fails to self-organize.

(B) Modelling boundary effects can affect the precise location of newly forming joints. Left and Right: Both weak and strong boundary effects can cause joints to divide existing phalanges (arrowhead). Middle: intermediate boundary effects bias joints to form near the distal tip (asterisk).

Development • Supplementary information

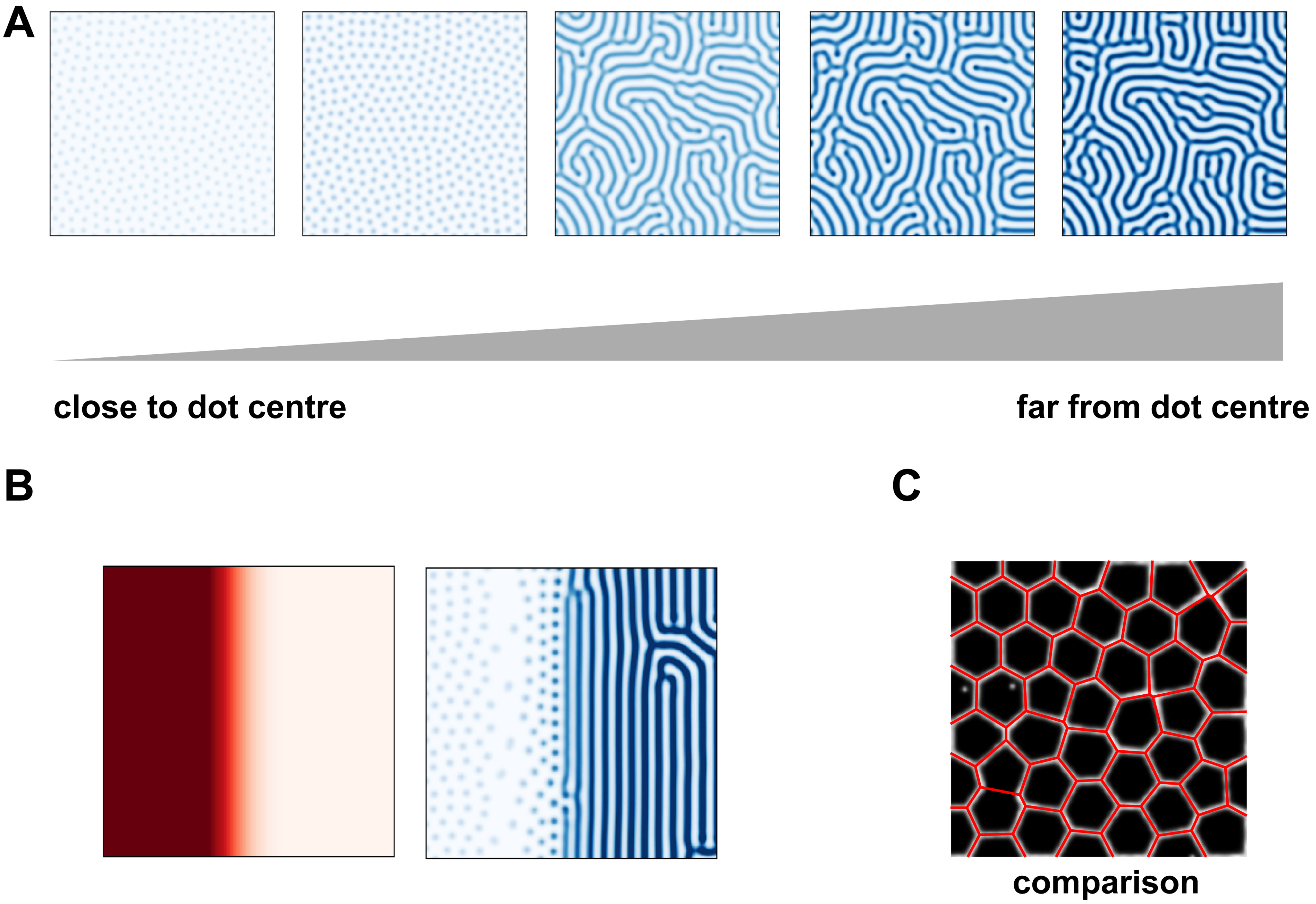

Figure S3

(A) Simulating the stripe system for uniform values of *A* and *S*, chosen to mimic being at different distances from a dot-centre in Fig. 4A.

(B) Simulating the stripe system for a one-dimensional gradient in the values of *A* and *S*, again chosen to mimic being at different distances from a dot-centre in Fig. 4A.

(C) Direct comparison of simulated joint patterns with the voronoi tessellation of Fig. 4A.

Development • Supplementary information

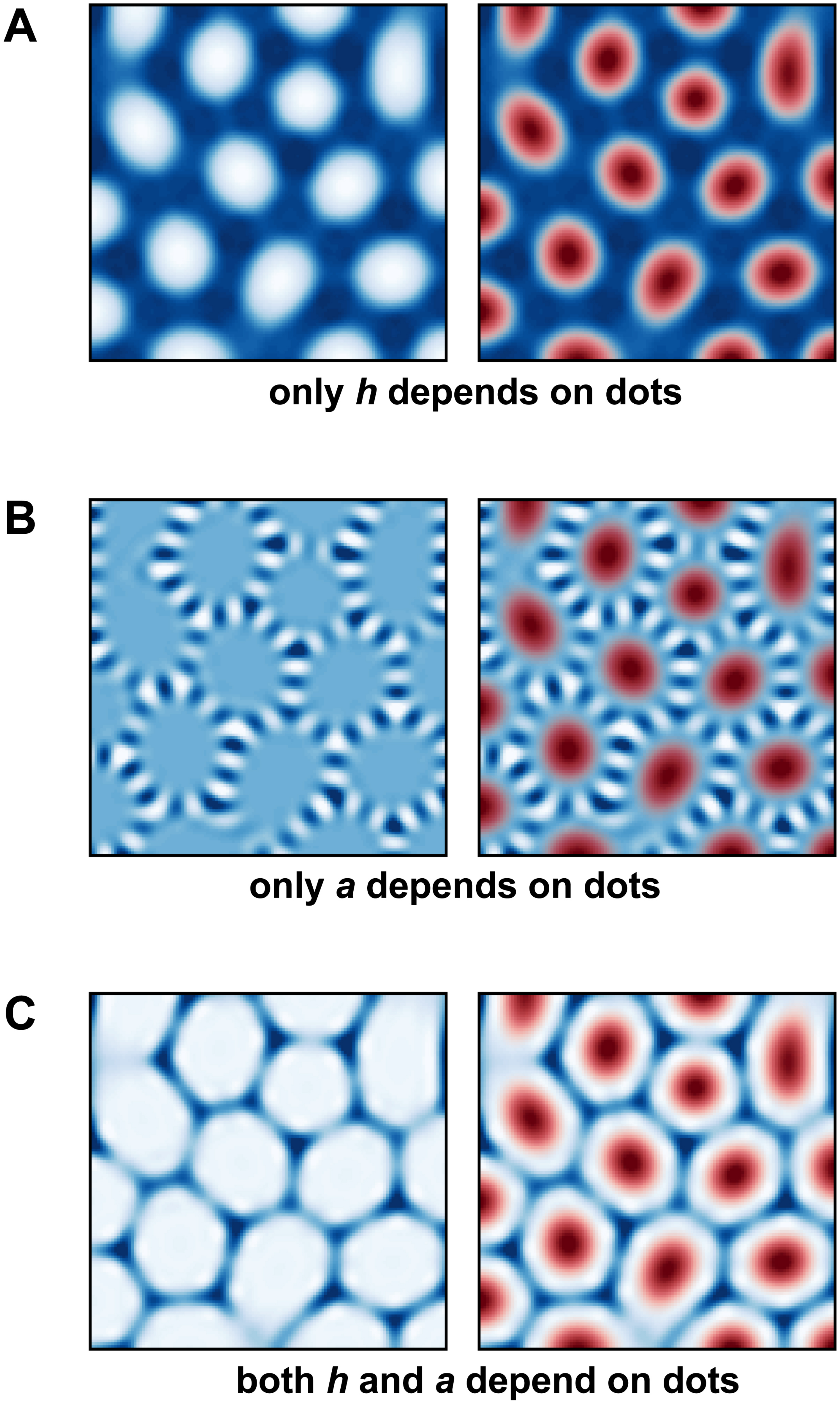

Development • Supplementary information

Figure S4

(A) Simulation of the generic dot-stripe system (Equations 7-8) with *h*-coupling only generates holes.

(B) Simulation of the generic dot-stripe system (Equations 7-8) with *a*-coupling only generates misoriented stripes.

(C) Simulation of the generic dot-stripe system (Equations 7-8) with both *h*- and *a*-coupling generates a polygonal lattice of joints.

Development • Supplementary information
