# Supplementary material for: A dot-stripe Turing model of joint patterning in the tetrapod limb
Source: Development. 2020 Apr 12;147(8):dev183699. doi: 10.1242/dev.183699 (PMC7174842; doi:10.1242/dev.183699)
Supplement: Reviewer comments [file develop-147-183699_review_history.pdf]

A dot-stripe Turing model of joint patterning in the tetrapod limb

Jake Cornwall Scoones and Tom W. Hiscock
DOI: 10.1242/dev.183699

Editor: Thomas Lecuit

Review timeline

Original submission:	12 August 2019
Editorial decision:	19 September 2019
First revision received:	4 December 2019
Editorial decision:	6 February 2020
Second revision received:	10 February 2020
Accepted:	24 February 2020

Original submission

First decision letter

MS ID#: DEVELOP/2019/183699

MS TITLE: A dot-stripe model of joint patterning in the tetrapod limb

AUTHORS: Jake Cornwall-Scoones and Tom Hiscock

I have now received all the referees' reports on the above manuscript, and have reached a decision. The referees' comments are appended below, or you can access them online: please go to BenchPressand click on the 'Manuscripts with Decisions' queue in the Author Area.

As you will see, the referees express considerable interest in your work, but have some significant criticisms and recommend a substantial revision of your manuscript before we can consider publication. There are three main issues that would need to be addressed:

1.Can the proposal of a coupled 2 Turing system rather a single network be justified? There is a suggestion that a single Turing-like network should be sufficient to produce similar behaviours. What would be the advantage of a 2-Turing network system and is there evidence to support this?

2. Where possible known features of the molecular/cellular mechanism of limb development, and the effect of mutations and perturbations, should be incorporated to constrain or support the model assumptions. This should include addressing the role of PFRs and whether the patterning mechanism is active throughout the whole forming digit or just at the distal tip. Illustrating the connections between theory and experimental observations is particularly important for Development.

3. Improve the explanation of the maths and improve the description of how growth affects model behaviour.

If you are able to revise the manuscript along the lines suggested, I will be happy receive a revised version of the manuscript. Your revised paper will be re-reviewed by one or more of the original referees, and acceptance of your manuscript will depend on your addressing satisfactorily the reviewers' major concerns. Please also note that Development will normally permit only one round of major revision.

© 2020. Published by The Company of Biologists under the terms of the Creative Commons Attribution License (https://creativecommons.org/licenses/by/4.0/).

Please attend to all of the reviewers' comments and ensure that you clearly highlight all changes made in the revised manuscript. Please avoid using 'Tracked changes' in Word files as these are lost in PDF conversion. I should be grateful if you would also provide a point-by-point response detailing how you have dealt with the points raised by the reviewers in the 'Response to Reviewers' box. If you do not agree with any of their criticisms or suggestions please explain clearly why this is so.

Reviewer 1

Advance summary and potential significance to field

In this manuscript, Cornwall-Scoones and Hiscock present a “generic” model for periodic digit phalanx-joint patterning using two coupled Turing mechanisms to generate patterns of phalanx condensations (dots) and interzone joint progenitors (stripes). Using hypothetical Turing networks without specifying molecular components, they focus on how variant periodic patterns can be generated and modulated by altering boundary and growth conditions to recapitulate patterns for wrist bones/joints, which are more lattice-like (polygonal), for mutants with altered digit joint patterns, and for vertebrate taxa with highly modified phalanx/joint patterns. Overall, I think the ideas presented are interesting and will stimulate further experimental work to test the fit and utility of the model.

Comments for the author

To be suitable for publication, the authors need to explain more clearly how/why their model is superior to a simpler single Turing interaction model. In addition, some of the background information on the digit phalanx/joint forming process is poorly summarized and inaccurate, and the math is presented in such a condensed fashion that it is very difficult to follow.

Specific points to consider:

1. The inclusion of 2 coupled Turing mechanisms to generate dots and stripes is appealing, but is it really necessary? Depending on boundary conditions and geometry, simple single activator-inhibitor Turing equations can generate dot or stripe patterns, so it seems possible that a single equation for dots could generate complementary “hole” patterns that become stripe-like. Can the authors demonstrate that the coupling of 2 Turing networks is superior to a single Turing mechanism for driving phalanx/dot formation and complementary holes/stripes specified by default? A demonstration, or a clearer explanation of the advantages of the 2-equation model would be helpful.

2. The authors formulate a generic model to avoid dealing with molecular mechanisms that may as yet be poorly understood, which is reasonable. But where features of the actual process are already known, this should be acknowledged up front and incorporated into the mechanism, and not over-simplified. Definitive lineage tracing studies in chick using defective retrovirus as well as descriptive marker evidence in mouse both indicate that the early digit ray is only a metapodial element (metacarpal or metatarsal), and phalanges do not arise from growth and subsequent segmentation of this single ray, but rather are generated from distal progenitors under the AER. These progenitors at the tip are distinct from the PFR (the incipient, newly-forming phalanx), and the new interzones (joint progenitors) arise simultaneously with the newly-forming PFR. However, throughout the introduction and the initial considerations for modeling in the results, the authors imply that phalanges arise via formation of interzones within an already existing digit ray.
Why not just acknowledge how the process occurs up front, and incorporate it into the Turing equations to begin with, since any model will need to be compatible with these observations?

3. Related to the above point, the authors indicate for the simulation shown in Figure 2, that a fast rate of growth is needed relative to the molecular interactions to achieve the addition of new phalanges/interzones (dots/stripes) from the tip rather than forming stripes within an existing digit ray. But intuitively, it seems unlikely that cell proliferation/growth rates would be fast relative to molecular diffusion rates. Can the authors clarify what constitutes “slow” or “fast”, and whether this is realistic in vivo? In mouse (Fig 2A), the time interval between successive stripes (Gdf5+) forming at the tip is about 12 hours.

© 2020. Published by The Company of Biologists under the terms of the Creative Commons Attribution License (https://creativecommons.org/licenses/by/4.0/).

Of interest, in the 5'Hoxd mutant, a situation where slower growth may occur, a pattern somewhat resembling a partial stripe specification occurs, however this never resolves to produce a stripe. Instead, Gdf5+ (stripe) descendants persist around the periphery of the site where a joint should have formed (see Huang et al, Fig 1M). Can the growth rate parameters also be adjusted to reproduce this type of joint loss?

4. Early and late events that are distinct developmentally are often conflated in the paper, which is confusing and misleading. Turing patterns to generate digit numbers (rays), and subsequent patterning of digit phalanges certainly could involve similar networks and both take cues from interdigit signals; however, they are distinct temporally. The digit rays, modeled by the Sharpe group with Turing equations, have already completely formed by E12 and are entirely metapodial; digit phalanges form subsequently, from tip progenitors as mentioned above, beginning around E12.5.
Likewise, the authors don't distinguish clearly between initial formation/induction of the interzone, the early joint anlage, and later joint maturation. For example, Schwartz et al. 2016 is cited (line 59) for showing interzones divide formed digits into phalanges, but this paper focuses on a continued recruitment of Gdf5+ cells following initial interzone formation. Confusion of early/late events is also evident in Table S1 (see comment below).

5. The math underlying the model presented is very tersely summarized in the Methods section – many parameters and other variables are not clearly explained. For example, "D" in equations 1 and 2 presumably refers to diffusion constants (not indicated anywhere), but what's the lowercase h in equation 1? Likewise, lowercase "k" usually signifies rate constants, but what is the lowercase "kappa" in equation 3b? In equation 7, "S" is presumably a hypothetical stripe molecule (not explicitly indicated), but is there an anti-stripe term analogous to "H" (for hole)? It would help to have a table organizing a list of all the terms and variables used and what they designate.
The table in the Methods (lines 592-3) lists parameter values used for different simulations in the figures, but how were these chosen? Was parameter space interrogated? How widely?

Minor points –

The categorizations of markers for dot/hole and stripe/antistripe are in some cases not entirely correct or somewhat arbitrary (Table S1).
Noggin is as much of a dot marker as Ihh – its expression arises within early condensations.
Bmpr1b (being the receptor through which Smad1,5 are activated) can also be viewed as a dot marker if pSmad1,5 are classified as dot genes. In fact, all of the anti-stripe markers can be viewed as dot/condensation markers. The distinction is not clear.
The stripe group includes early (interzone) and late stage differentiating joint markers (eg. tenascin; expressed after all interzones have already formed) – only the early markers are relevant to the early dot/stripe induction events being modeled.

Similarly, Col10a1 is expressed at a much later stage, in hypertrophic cartilage, and furthermore is within elongating condensations, NOT in interzones or early joints, and so is not a stripe marker at all.

In the introduction, the description of limb components: stylopod/zeugopod as (arm/leg) is confusing; I think something like: (proximal/distal long bones of limb) would be clearer in meaning.

<u>Reviewer 2</u>

Advance summary and potential significance to field

The authors construct a phenomenological four-component reaction-diffusion model to match observed digit joint and phalanx patterns, with the joints being "stripes" of expression across each digit organised by one Turing morphogen pair and the "dots" being the phalanges organised by another pair with a minimal inhibitory interaction between the pairs. Their model fits the longitudinal stripes previously seen for a joint marker in the Jaws mouse mutant (a "generalised" ECM integrity mutant thought to affect long-range Ihh signalling). It also fits the limbs of some fossil species with many phalanges per digit.

© 2020. Published by The Company of Biologists under the terms of the Creative Commons Attribution License (https://creativecommons.org/licenses/by/4.0/).

Comments for the author

This paper is fine as far as it goes, but resembles many of the theoretical papers on RD patterning from the 1970s and '80s: biological patterns can be very plausibly matched with RD models, but in fact too easily. (The quotation is "give me three parameters and I can make an elephant; give me four and I can make it wiggle its tail"). With four morphogens there are too many parameters and so the modelling is not telling us very much. More specifically, there is not much justification for invoking two separate morphogen pairs for dots and stripes, and it could well be that with a bit more effort the same patterns could be achieved with just two morphogens (with the dots being merely the spaces between the stripes – see for example the white areas in the right-most panel in fig. 5B). Even the "counterituitive" longitudinal stripes seen in the Jaws mutation (previously reported by the Harland lab, so not original in this submission) can be produced by parameter changes in a two-component system (see, e.g., Asai et al., 1999, Mech. Dev. Fig. 3).

Thus, while what this paper describes is probably broadly correct, it falls between the two stools of being too complex (too many parameters) and too simple (generic, phenomenological, under-constrained).

<u>Reviewer 3</u>

Advance summary and potential significance to field

Cornwall-Scoones and Hiscock propose a novel phenomenological model of joint patterning during tetrapod limb development. The model couples two Turing models: a dot-forming and a stripe-forming Turing model. In this way, the model can recapitulate four types of periodic patterns that can be associated with four broad classes of expression patterns observed during digit development:
Class 1a patterns - stripes that mark the forming joints, Class 1b patterns -anti-stripes that mark the digit, Class 2a patterns - dot that mark condensations at the center of each phalanx, Class 2b patterns - anti-dot that are opposite to condensations. The authors show that this model can explain the patterns observed in Jaws mutants. Finally they propose that modulations of this joint-patterning model could explain the formation of different types of skeletal elements during the evolution of tetrapod appendage.

Comments for the author

Cornwall-Scoones and Hiscock are proposing an interesting new hypothetical model of phalanx patterning. They state that the goal of the model isn't to explain specific molecular joint markers, but rather to provide a phenomenological explanation for the qualitative patterns associated with joint formation. I have no problem with this posture, but I am concerned by the lack of support for the specific model choice that they are proposing. Indeed, the authors don't present enough evidence to support a model that couples two Turing mechanisms. For this reason, I have the impression that in his current form, this study does not improve our understanding of phalanx patterning. I also find surprising that the model does not account for the formation of PFR/DC at digit tips and that it cannot intrinsically explain the smaller progressive length of distal phalanges. Discussing additional mutants could help support the model further.

Specific points
a) PATTERNS: The authors develop a model that couples a dot-forming Turing pattern and stripe-forming Turing pattern to explain the formation of Class Ia and Ib (stripes and anti-stripes) and Class 2a and 2b (dots and anti-dots).
However, the model could explain only the earliest patterning events in phalanx formation. These include indeed the formation of Ia and Ib patterns but I don't see the need for dot or anti-dot patterns in the context of phalanx patterning. Dot-patterns appear later and reflect condensations events within digits/phalanx which are not necessarily related to phalanx patterning. The authors should analyze in more detail the literature looking for the temporal expression of the different factors presented in Table S1, making sure that their self-organizing models focus only on early events. If dot patterns are ignored, a much simpler stripe-forming substrate-depleted Turing model

© 2020. Published by The Company of Biologists under the terms of the Creative Commons Attribution License (https://creativecommons.org/licenses/by/4.0/).

can actually explain the formation of Ia and Ib patterns simultaneously without the need of a second Turing model. Do they really need to explain four different patterns of stripes, anti-stripes, dot patterns and dot-holes? Even if they need to explain only stripe and dot-patterns, a single substrate-depleted Turing model that forms spots is enough, since it will form spots for on factor and stripes for the other. This is the case for example in the dot-forming Turing system that they present in Figure 1C, that make dots for the red factor and stripes for the green factor. Overall, it is surprising that they focus their model on dot and anti-dot patterns and for example they ignore the formation of distal structures like PFRs/DCs.

b) BOUNDARY: A very important ingredient of their model is the boundary condition that favour the formation of stripes that are aligned along the boundary of the digit. In the absence of this boundary condition the joint patterns would be disorganized as in the case of human wrist or icthythosaur paddles. Can they find any biological evidence for this boundary in mouse? Are there mutants that show a lack of influence from the boundary? The jaw mutant that they present in Figure 3B for example shows disorganized joint patterns, can these patterns be interpreted as a loss of boundary influence rather than a difference in diffusivity of the dot-forming molecule? A more extensive biological and theoretical discussion on boundary conditions should be included in the study. One possibility is that there are no boundary conditions and that the joint pattern is aligned due to the narrower spatial domain of the the digit, as they show in the narrow simulation in figure 5B. Would a narrow domain be a robust strategy to align joints? Is there any mutant that has larger digits with disorganized joint stripe patterns?

c) GROWTH:
In Figure 2 they discuss the effect of digit growth on the model and show that for slow growth the new joints appear by division of the proximal phalanx rather than by addition of a phalanx at the distal tip. This show an intrinsic proximal phalanx plasticity that has never been observed in the real biological systems and it appear that any change in digit growth speed would uncover it. Is there any mutant where lower growth speed (E.g. lower Fgf signal) lead to splitting more proximal phalanges? They also don't provide enough details of how they are modeling growth. Are they just stretching the whole digit distally? In alternative, would a distal accretion growth make the system more robust to growth change?

Minor point: They should avoid the use of the term "joint molecules" in the abstract as it can be confusing they can instead use "molecular joint markers".

First revision

<u>Author response to reviewers' comments</u>

<u>Reviewer 1</u>
Advance Summary and Potential Significance to Field:
In this manuscript, Cornwall-Scoones and Hiscock present a “generic” model for periodic digit phalanx-joint patterning using two coupled Turing mechanisms to generate patterns of phalanx condensations (dots) and interzone joint progenitors (stripes). Using hypothetical Turing networks without specifying molecular components, they focus on how variant periodic patterns can be generated and modulated by altering boundary and growth conditions to recapitulate patterns for wrist bones/joints, which are more lattice-like (polygonal), for mutants with altered digit joint patterns, and for vertebrate taxa with highly modified phalanx/joint patterns. Overall, I think the ideas presented are interesting and will stimulate further experimental work to test the fit and utility of the model.

> We are grateful to the reviewer for their helpful comments and suggestions, and hope that the edits outlined below have significantly improved the manuscript. We share the reviewer’s enthusiasm for using mathematical models to stimulate and guide experimental work.

© 2020. Published by The Company of Biologists under the terms of the Creative Commons Attribution License (https://creativecommons.org/licenses/by/4.0/).

Reviewer 1 Comments for the Author:
To be suitable for publication, the authors need to explain more clearly how/why their model is superior to a simpler single Turing interaction model. In addition, some of the background information on the digit phalanx/joint forming process is poorly summarized and inaccurate, and the math is presented in such a condensed fashion that it is very difficult to follow.

Specific points to consider:

1. The inclusion of 2 coupled Turing mechanisms to generate dots and stripes is appealing, but is it really necessary? Depending on boundary conditions and geometry, simple single activator-inhibitor Turing equations can generate dot or stripe patterns, so it seems possible that a single equation for dots could generate complementary "hole" patterns that become stripe-like. Can the authors demonstrate that the coupling of 2 Turing networks is superior to a single Turing mechanism for driving phalanx/dot formation and complementary holes/stripes specified by default? A demonstration, or a clearer explanation of the advantages of the 2-equation model would be helpful.

This is an excellent suggestion; the justification of combining dot- and stripe-forming systems was indeed lacking in our original manuscript, and we have taken several steps to remedy this in our revision.

First, we are encouraged that the reviewer raised the hypothesis of a single Turing system for joint patterning comprising dots and holes – which we refer to as a dot- hole Turing system. Starting out on this project, we thought that the most natural / simple model to form joints would be a stripe-forming Turing system, since joint patterns in wildtype limbs resemble stripe-like patterns (in fact, reviewer 3 suggested that we consider a stripe-only model). Therefore we think that an important part of this manuscript is the (perhaps counterintuitive) proposal that there must be a dot-forming Turing system operative during joint patterning to explain aberrant joint morphologies observed in different mutants and species. In this regard, we share the reviewer's intuition that a dot-forming system is the central feature of our model.

However, we would argue that whilst a dot-hole system can explain the correct topology of joint patterning in both wildtype and mutant limbs, such a model fails to capture the local structure and shape of joints. More specifically, a dot-hole system would produce joints that are wide, curved and not straight. We suggest that it is the combination of a dot-forming system (that controls the overall topology of the pattern) with a stripe-forming system (that ensures joints are narrow and straight) which allows the dot-stripe mechanism to reproduce in vivo joint patterns.

To demonstrate this point clearly, we have included a new section in the manuscript (lines 349-379), together with a new figure (Figure 5), in which we have included side-by-side simulations of dot-hole, stripe-only and dot-stripe mechanisms to allow direct comparison of the different models. We hope that this clearly demonstrates our reasons for considering a combined dot-stripe model. We have also tried to clarify our presentation of dot-, hole- and stripe-like gene expression patterns from the literature; referred to in lines 124-139 and (a revised) Table S1.

2. The authors formulate a generic model to avoid dealing with molecular mechanisms that may as yet be poorly understood, which is reasonable. But where features of the actual process are already known, this should be acknowledged up front and incorporated into the mechanism, and not over-simplified. Definitive lineage tracing studies in chick using defective retrovirus as well as descriptive marker evidence in mouse both indicate that the early digit ray is only a metapodial element (metacarpal or metatarsal), and phalanges do not arise from growth and subsequent segmentation of this single ray, but rather are generated from distal progenitors under the AER. These progenitors at the tip are distinct from the PFR (the incipient, newly-forming phalanx), and the new interzones (joint progenitors) arise simultaneously with the newly-forming PFR. However, throughout the introduction and the initial considerations for modeling in the results, the authors imply that phalanges arise via formation of interzones within an already existing digit ray.
Why not just acknowledge how the process occurs up front, and incorporate it into the Turing

© 2020. Published by The Company of Biologists under the terms of the Creative Commons Attribution License (https://creativecommons.org/licenses/by/4.0/).

equations to begin with, since any model will need to be compatible with these observations?

We apologize for this shortcoming – it was not our intention in the introduction to imply that interzones form within the main body of an existing digit ray. We have amended the relevant sections as suggested (see lines 60-64), and hope that this clarifies things.

In the case of the mathematical modelling, we think it still makes sense for us to build up the model gradually, starting out simple and progressively adding more complexity and realism. In particular, the advantage of starting out with a fixed geometry is that we can explore the core self-organizing properties of the dot-stripe model, and optimize parameters, before then introducing growth. We have explained this rationale in the main text (in lines 189-194).

We also realize that we may not have clearly explained how we were modelling digit ray growth in our simulations – we were considering elongation just at the very tip, similar to your suggestion. We have amended Figure 2B accordingly.

In the same spirit of adding layers of complexity/realism– and in response to comments from other reviewers – we have incorporated other processes into our model that may be operative in vivo. In Figure 2C and lines 242-249, we describe simulations that demonstrate that the dot-stripe mechanism need only operate towards the distal end of the digit ray for normal patterning to occur. This result can be interpreted through the lens of a phalanx-forming region at the distal tip.
Furthermore, in Figure 2D and lines 251-258, we consider the effect of adding uniform growth along the digit (in addition to the distal elongation from sub-AER progenitor cells). In this scenario, phalanges progressively decrease in size from proximal to distal, thereby providing a hypothesis to explain the variation in phalangeal proportions along PD observed in many different species.

3. Related to the above point, the authors indicate for the simulation shown in Figure 2, that a fast rate of growth is needed relative to the molecular interactions to achieve the addition of new phalanges/interzones (dots/stripes) from the tip rather than forming stripes within an existing digit ray. But intuitively, it seems unlikely that cell proliferation/growth rates would be fast relative to molecular diffusion rates. Can the authors clarify what constitutes "slow" or "fast", and whether this is realistic in vivo? In mouse (Fig 2A), the time interval between successive stripes (Gdf5+) forming at the tip is about 12 hours.

We thank the reviewer for highlighting this point and potential cause for confusion. Firstly, in our original draft, we presented the two simulations as having "fast" or "slow" growth, relative to molecular patterning kinetics. The way we implemented this in our simulations was by changing the overall timescale of the simulations, but keeping the initial and final digit sizes the same. A longer overall timescale can be interpreted as either slower growth relative to patterning kinetics, or faster patterning kinetics relative to growth. We realize that, since digit growth can then be directly measured whereas the rate constants in our model are unknown, it makes more sense to explain the results assuming a constant growth rate and varying patterning kinetics. Therefore, "fast/slow growth" is better written as "slow/fast patterning kinetics". We have changed Figure S2A and lines 260-268 accordingly and hope that this makes more sense.

Having said this, as you suggest, we do not have quantitative data for patterning kinetics (i.e. transcription/degradation/diffusion rates) and so we cannot directly test whether our model correctly predicts nascent GDF5 expression at the distal tip for measured parameters. However, we would disagree with your suggestion that it is unreasonable for the patterning kinetics to be of the right magnitude (i.e. that they are too fast compared to growth) for this to be the case. One reason is that we actually find that if patterning kinetics are either too fast or two slow, then this disrupts the emergence of stripes at the distal tip (Figure S2A, right). Therefore, it is an intermediate value of patterning kinetics in our model that is required. Secondly, for this intermediate speed (in Figure S2A, middle), we can make a very rough estimate of some of the parameters in our model and this estimate does not seem wildly unreasonable. (We do not expect our parameter estimates to

© 2020. Published by The Company of Biologists under the terms of the Creative Commons Attribution License (https://creativecommons.org/licenses/by/4.0/).

be quantitatively correct, but only in the right magnitude.) For example, if we assume that the timescale between adjacent joints to form is about 12 hrs, and then focusing on the dot-forming molecule as an example, we predict its half-life (ka^{-1}) to be around 0.5 hrs.

A quantitative assessment of our model – including more detailed quantitative measurements on in vivo patterning dynamics – remains outside the scope of our current work. In our revision, we have emphasized the parameter sensitivity of our conclusions (lines 274-277), as well as moving this set of results to supplementary (Fig S2A). In addition, and in response to comments from another reviewer, we considered other effects that may bias stripe expression towards the very distal tip such as boundary effects (lines 268-274 and Fig S2B).

Of interest, in the 5'Hoxd mutant, a situation where slower growth may occur, a pattern somewhat resembling a partial stripe specification occurs, however this never resolves to produce a stripe. Instead, Gdf5+ (stripe) descendants persist around the periphery of the site where a joint should have formed (see Huang et al, Fig 1M). Can the growth rate parameters also be adjusted to reproduce this type of joint loss?

This is a very interesting observation. Whilst we haven't observed such a phenotype by altering growth rates in silico, it seems possible that other parameter changes could generate such a behaviour. Intuitively, the peripheral regions are those which are furthest from the "dots" and thus perhaps stripe patterning could be initiated in these regions but fail to connect and/or persist to form full stripes. Understanding this mutant would be an interesting extension and application of our model, but we have not directly considered it in this manuscript.

4. Early and late events that are distinct developmentally are often conflated in the paper, which is confusing and misleading. Turing patterns to generate digit numbers (rays), and subsequent patterning of digit phalanges certainly could involve similar networks and both take cues from interdigit signals; however, they are distinct temporally. The digit rays, modeled by the Sharpe group with Turing equations, have already completely formed by E12 and are entirely metapodial; digit phalanges form subsequently, from tip progenitors as mentioned above, beginning around E12.5.

We agree with this point, and have modified sections of the text accordingly.

Likewise, the authors don't distinguish clearly between initial formation/induction of the interzone, the early joint anlage, and later joint maturation. For example, Schwartz et al. 2016 is cited (line 59) for showing interzones divide formed digits into phalanges, but this paper focuses on a continued recruitment of Gdf5+ cells following initial interzone formation. Confusion of early/late events is also evident in Table S1 (see comment below).

We apologize for this incorrect reference - we originally cited Schwartz et al 2016 referring to its introduction rather than main results. We have altered the reference to a more appropriate review paper (lines 59; reference: Decker, R.S., Koyama, E., and Pacifici, M., 2014. Genesis and morphogenesis of limb synovial joints and articular cartilage. Matrix Biology 39 (2014): 5-10.).

5. The math underlying the model presented is very tersely summarized in the Methods section – many parameters and other variables are not clearly explained. For example, "D" in equations 1 and 2 presumably refers to diffusion constants (not indicated anywhere), but what's the lowercase h in equation 1? Likewise, lowercase "k" usually signifies rate constants, but what is the lowercase "kappa" in equation 3b? In equation 7, "S" is presumably a hypothetical stripe molecule (not explicitly indicated), but is there an anti-stripe term analogous to "H" (for hole)? It would help to have a table organizing a list of all the terms and variables used and what they designate.
The table in the Methods (lines 592-3) lists parameter values used for different simulations in the figures, but how were these chosen? Was parameter space interrogated? How widely?

We are grateful to the reviewer for raising these issues and have made efforts to describe

© 2020. Published by The Company of Biologists under the terms of the Creative Commons Attribution License (https://creativecommons.org/licenses/by/4.0/).

our modelling methods more clearly, and hope that this has clarified our approach. In particular, we have changed notation, as suggested, and have more fully described all variables and parameters (see Table S2). We have also described how we chose parameters for simulations (lines 654-666).

Minor points –

The categorizations of markers for dot/hole and stripe/antistripe are in some cases not entirely correct or somewhat arbitrary (Table S1).
Noggin is as much of a dot marker as Ihh – its expression arises within early condensations. Bmpr1b (being the receptor through which Smad1,5 are activated) can also be viewed as a dot marker if pSmad1,5 are classified as dot genes. In fact, all of the anti-stripe markers can be viewed as dot/condensation markers. The distinction is not clear.
The stripe group includes early (interzone) and late stage differentiating joint markers (eg. tenascin; expressed after all interzones have already formed) – only the early markers are relevant to the early dot/stripe induction events being modeled.
Similarly, Col10a1 is expressed at a much later stage, in hypertrophic cartilage, and furthermore is within elongating condensations, NOT in interzones or early joints, and so is not a stripe marker at all.

We have substantially altered Table S1, motivated by these comments, and have only included genes for which we can find evidence that they are expressed early in patterning (i.e. within a newly-forming, distally-located phalanx). Moreover, we have modified our classes of gene expression patterns to "Dot", "Hole" and "Stripe" to more accurately reflect these early expression patterns, omitting "anti-stripe" patterns which are not as apparent. Finally we have included a column describing phenotypes corresponding to perturbations of the given genes.

In the introduction, the description of limb components: stylopod/zeugopod as (arm/leg) is confusing; I think something like: (proximal/distal long bones of limb) would be clearer in meaning.

Yes, this was confusing! We have modified lines 27 accordingly.

<u>*Reviewer 2*</u>
Advance Summary and Potential Significance to Field:
The authors construct a phenomenological four-component reaction-diffusion model to match observed digit joint and phalanx patterns, with the joints being "stripes" of expression across each digit organised by one Turing morphogen pair and the "dots" being the phalanges organised by another pair with a minimal inhibitory interaction between the pairs. Their model fits the longitudinal stripes previously seen for a joint marker in the Jaws mouse mutant (a "generalised" ECM integrity mutant thought to affect long-range Ihh signalling). It also fits the limbs of some fossil species with many phalanges per digit.

We thank the reviewer for their comments and hope our revised manuscript has gone some way in addressing the concerns raised. Our aim is to formulate a model that generates new hypotheses for joint patterning and stimulates further experimental work, and sincerely believe that this is the case.

Reviewer 2 Comments for the Author:
This paper is fine as far as it goes, but resembles many of the theoretical papers on RD patterning from the 1970s and '80s: biological patterns can be very plausibly matched with RD models, but in fact too easily. (The quotation is "give me three parameters and I can make an elephant; give me four and I can make it wiggle its tail"). With four morphogens there are too many parameters and so the modelling is not telling us very much.

Whilst we agree, in general, that there is a risk of overfitting overly-complex models to data, we do not think that this is the case for the dot-stripe model presented in this

© 2020. Published by The Company of Biologists under the terms of the Creative Commons Attribution License (https://creativecommons.org/licenses/by/4.0/).

manuscript.

Firstly, we acknowledge that there are many parameters in our model. However, when comparing predictions of our model, most of these parameters are fixed based on their ability to generate wildtype patterns. For example, in Figure 3, we are only varying two free parameters to generate the mutant phenotypes; and in Figures 4-5 we obtain a lattice-like morphology simply by changing a single parameter: the width of the digit domain.

Second, in our original manuscript, we did try to reduce the number of parameters in our model. In particular, we showed that a simpler description of the dot-stripe mechanism (using coupled Swift Hohenberg equations, Fig 6) could capture the same phenomenology of patterns as the more complex dot-stripe model introduced in Figure 1.

More specifically, there is not much justification for invoking two separate morphogen pairs for dots and stripes, and it could well be that with a bit more effort the same patterns could be achieved with just two morphogens (with the dots being merely the spaces between the stripes – see for example the white areas in the right-most panel in fig. 5B).

This is an excellent point, and we note that the justification of combining two separate morphogen pairs for dot- and stripe-forming systems was lacking in our original manuscript. We have taken several steps to remedy this in our revision, and have added a new section (lines 349-379) and new simulation results (Figure 5).

Starting out on this project, we thought that the most natural / simple model to form joints would be a stripe-forming Turing system, since joint patterns in wildtype limbs resemble stripe-like patterns - reviewer 3 suggested that we consider a stripe-only model. However, when we simulated such a model, we found that whilst it could recapitulate wildtype joint patterns, it failed to correctly predict mutant phenotypes (Figure 5A).

Similarly, as you and reviewer 1 suggest, we considered a model involving a single morphogen pair that generates stripey-hole patterns, corresponding to joints, separated by dots. Whilst such a dot-hole system can explain the correct topology of joint patterning in both wildtype and mutant limbs, such a model fails to capture the local structure and shape of joints. More specifically, a dot-hole system produces joints that are wide, curved and not straight (Figure 5B). We suggest that it is the combination of a dot-forming system (that controls the overall topology of the pattern) with a stripe-forming system (that ensures joints are narrow and straight) that allows the dot-stripe mechanism to explain in vivo joint patterns (Figure 5C).

We have also tried to clarify our presentation of expression patterns from the literature, which involves genes that have demonstrated roles in joint patterning and clearly show dot, hole or stripe patterns (referred to in lines 124-139 and a revised Table S1). This provides further justification for considering both dot-forming and stripe-forming systems.

Even the "counterituitive" longitudinal stripes seen in the Jaws mutation (previously reported by the Harland lab, so not original in this submission) can be produced by parameter changes in a two-component system (see, e.g., Asai et al., 1999, Mech. Dev. Fig. 3).

We thank the reviewer for highlighting this interesting paper, although, for the reasons outlined above, maintain that there is good justification for combining dot- and stripe-systems. Moreover, we note that the simulations from Asai et al 1999 involve inhomogeneous initial conditions ("a random pattern with three thin lines") and dynamic changes of system parameters ("diffusion constants are linearly decreased"), assumptions that we do not consider in this manuscript.

Thus, while what this paper describes is probably broadly correct, it falls between the two stools of being too complex (too many parameters) and too simple (generic, phenomenological, under-constrained).

© 2020. Published by The Company of Biologists under the terms of the Creative Commons Attribution License (https://creativecommons.org/licenses/by/4.0/).

Reviewer 3
Advance Summary and Potential Significance to Field:
Cornwall-Scoones and Hiscock propose a novel phenomenological model of joint patterning during tetrapod limb development. The model couples two Turing models: a dot-forming and a stripe-forming Turing model. In this way, the model can recapitulate four types of periodic patterns that can be associated with four broad classes of expression patterns observed during digit development: Class 1a patterns - stripes that mark the forming joints, Class 1b patterns - anti-stripes that mark the digit, Class 2a patterns - dot that mark condensations at the center of each phalanx, Class 2b patterns - anti-dot that are opposite to condensations. The authors show that this model can explain the patterns observed in Jaws mutants. Finally they propose that modulations of this joint-patterning model could explain the formation of different types of skeletal elements during the evolution of tetrapod appendage.

We thank the reviewer for their comments and suggestions, and hope that our efforts to address these have significantly improved our revised manuscript.

Reviewer 3 Comments for the Author:
Cornwall-Scoones and Hiscock are proposing an interesting new hypothetical model of phalanx patterning. They state that the goal of the model isn't to explain specific molecular joint markers, but rather to provide a phenomenological explanation for the qualitative patterns associated with joint formation. I have no problem with this posture, but I am concerned by the lack of support for the specific model choice that they are proposing. Indeed, the authors don't present enough evidence to support a model that couples two Turing mechanisms.

We agree that the justification of coupling two Turing mechanism – one dot-forming and one stripe-forming – was lacking in our original manuscript, and have taken several steps to remedy this in our revision which we outline below.

Starting out on this project, we thought that the most natural / simple model to form joints would be a Turing stripe-forming system, since joint patterns in wildtype limbs resemble stripe-like patterns (as suggested in your comments below). However, when we simulated such a model, we found that whilst it could recapitulate wildtype joint patterns, it failed to correctly predict mutant phenotypes (Figure 5A).

Similarly, we considered a single dot-hole Turing mechanism that generates stripey- hole patterns, corresponding to joints, separated by dots. Whilst such a dot-hole system can explain the correct topology of joint patterning in both wildtype and mutant limbs, such a model fails to capture the local structure and shape of joints. More specifically, a dot-hole system produces joints that are wide, curved and not straight (Figure 5B). We suggest that it is the combination of a dot-forming system (that controls the overall topology of the pattern) with a stripe-forming system (that ensures joints are narrow and straight) that allows the dot-stripe mechanism to explain in vivo joint patterns (Figure 5C). To demonstrate this point clearly, we have included a new section in the manuscript (lines 349-379), together with a new figure (Figure 5), in which we have included side-by-side simulations of dot-hole, stripe- only and dot-stripe mechanisms to allow direct comparison of the different models. We hope that this clearly demonstrates our reasons for considering two coupled Turing systems.

We have also tried to clarify our presentation of expression patterns from the literature, which involves genes that have demonstrated roles in joint patterning and clearly show dot, hole or stripe patterns (referred to in lines 124-139 and a revised Table S1). This provides further justification for considering both dot-forming and stripe-forming systems.

For this reason, I have the impression that in his current form, this study does not improve our understanding of phalanx patterning. I also find surprising that the model does not account for the formation of PFR/DC at digit tips and that it cannot intrinsically explain the smaller progressive length of distal phalanges.
Discussing additional mutants could help support the model further.

In response to these suggestions, we have performed several new simulations and have

© 2020. Published by The Company of Biologists under the terms of the Creative Commons Attribution License (https://creativecommons.org/licenses/by/4.0/).

included these in the revised version of our manuscript.

Firstly, we considered the case where patterning via the dot-stripe mechanism is not occurring throughout the entire digit ray, but rather only in a restricted distal region (akin to the 'phalanx forming region'). In this scenario, we thus assume that once cells are sufficiently proximal such that they leave this region, then their joint/phalanx fates are fixed (i.e. the molecular concentrations of the model variables are held constant). Under these conditions, we find the dot-stripe model can still reproduce observed joint patterns, as long as the size of the patterning region is at least as large as the typical phalanx size. We provide details of these results in lines 242-249 and Figure 2C.

A second characteristic of the PFR/DC is a localized "crescent" of BMP activity around the distal tip of the elongating digit ray (see Montero et al. 2008). We wondered what effect that this might have on joint patterning in our model. When we simulated such a boundary term, we found that nascent stripe expression could be biased towards the distal tip, as is observed in vivo. We have included a discussion of these simulations in lines 268-274 and Figure S2B.

Finally, in relation to the "smaller progressive length of distal phalanges", we had an intuition that growth may be playing a role, since proximal phalanges are formed first and may be larger simply by virtue of having had more time to lengthen. To test this intuition, we combined elongation of the digit at its tip (see also our response to your comment below) with uniform growth along the digit, and found that this could naturally explain phalanges of decreasing distal proportion. This provides one hypothesis for why many species display this variation in phalanx size.

Specific points
a) PATTERNS: The authors develop a model that couples a dot-forming Turing pattern and stripe-forming Turing pattern to explain the formation of Class Ia and Ib (stripes and anti-stripes) and Class 2a and 2b (dots and anti-dots). However, the model could explain only the earliest patterning events in phalanx formation. These include indeed the formation of Ia and Ib patterns but I don't see the need for dot or anti-dot patterns in the context of phalanx patterning. Dot-patterns appear later and reflect condensations events within digits/phalanx which are not necessarily related to phalanx patterning. The authors should analyze in more detail the literature looking for the temporal expression of the different factors presented in Table S1, making sure that their self-organizing models focus only on early events.

We have substantially altered Table S1, motivated by these comments, and have only included genes that are expressed early in patterning - defined to be within a newly-forming, distally-located phalanx. (We note that dot patterns are evident early during phalanx patterning – see BMP activity in Huang et al 2016 or Ihh/Ppr in Gao et al 2009 for examples.) Moreover, we have modified our classes of gene expression patterns to "Dot", "Hole" and "Stripe" to more accurately reflect these early expression patterns, omitting "anti-stripe" patterns which are not as apparent. Finally we have included a column describing phenotypes corresponding to perturbations of the given genes.

If dot patterns are ignored, a much simpler stripe-forming substrate-depleted Turing model can actually explain the formation of Ia and Ib patterns simultaneously without the need of a second Turing model.

At the outset of this project, we shared this intuition – a stripe-only model seems the most natural way to explain regularly spaced joints in wildtype digits. However, as discussed in detail above, we have included new results that demonstrate why a stripe-only Turing system fails to explain the diversity of joint morphologies observed in vivo (Figure 5).

Do they really need to explain four different patterns of stripes, anti-stripes, dot patterns and dot-holes? Even if they need to explain only stripe and dot-patterns, a single substrate- depleted Turing model that forms spots is enough, since it will form spots for on factor and stripes for the other. This is the case for example in the dot-forming Turing system that they present in Figure 1C, that make dots for the red factor and stripes for the green factor.

© 2020. Published by The Company of Biologists under the terms of the Creative Commons Attribution License (https://creativecommons.org/licenses/by/4.0/).

Again, we (and the other reviewers) share this intuition. However, as discussed in detail above, our new simulation results suggest that a dot-hole Turing system cannot adequately capture joint shape (narrow and straight, Figure 5B), and instead a combination of dot- and stripe-forming systems is required (Figure 5C).

Overall, it is surprising that they focus their model on dot and anti-dot patterns and for example they ignore the formation of distal structures like PFRs/DCs.

As discussed above, we have added new simulations to address this point (Figure 2 and Figure S2).

b) BOUNDARY: A very important ingredient of their model is the boundary condition that favour the formation of stripes that are aligned along the boundary of the digit.

We agree that the boundary conditions are certainly important to our model, and have expanded our discussion of this in lines 208-213 in an attempt to more accurately explain and justify our choice.

In the absence of this boundary condition the joint patterns would be disorganized as in the case of human wrist or icthythosaur paddles. Can they find any biological evidence for this boundary in mouse? Are there mutants that show a lack of influence from the boundary?

Indeed, our simulations predict a disorganization of the joint patterns in the absence of our chosen boundary conditions, in which the dot-molecule is degraded outside of the digit (Figure S1C). We speculate that this repression/degradation could be being mediated by mesenchymal/perichondrial cells that flank the digits, however we can find no direct evidence for this. Therefore, we have to assume that such a boundary condition exists; in the absence of experimental data, this remains an assumption, but one that could be experimentally tested if a putative dot-forming molecule were found.

The jaw mutant that they present in Figure 3B for example shows disorganized joint patterns, can these patterns be interpreted as a loss of boundary influence rather than a difference in diffusivity of the dot-forming molecule?

This is a nice idea, but when we varied the strength of the boundary term in Figure S1C, we saw a general disorganization of joint patterns, rather than new joints forming along the digit length as observed in the Jaws mutant. Therefore we think these mutants are better explained by differences in dot-molecule diffusivities.

A more extensive biological and theoretical discussion on boundary conditions should be included in the study. One possibility is that there are no boundary conditions and that the joint pattern is aligned due to the narrower spatial domain of the the digit, as they show in the narrow simulation in figure 5B. Would a narrow domain be a robust strategy to align joints?

Yes! We may not have been clear in our first draft – the lattice-like patterns in Figure 4 have the same boundary conditions as the digit simulations in Figures 1-3, but just on a different (wider) geometry. Therefore our results suggest that a narrow domain is a robust strategy to align joints, provided suitable boundary terms are modelled.

Figure S1C demonstrates that a narrow domain is not sufficient alone to align joints. We have modified text lines 311-312 to emphasize this point.

Is there any mutant that has larger digits with disorganized joint stripe patterns?

We have not come across any clear examples of such a phenotype, but would be very interested to find them! The closest we found are reports of cetacean flippers in which multiple digits fuse within the autopod; in these regions, joint alignment appears disrupted. See Figure 3 in:

© 2020. Published by The Company of Biologists under the terms of the Creative Commons Attribution License (https://creativecommons.org/licenses/by/4.0/).

Cooper, L.N., Dawson, S.D., 2009. The trouble with flippers: a report on the prevalence of digital anomalies in Cetacea. Zoological Journal of the Linnean Society 155, 722-735.

c) GROWTH:
In Figure 2 they discuss the effect of digit growth on the model and show that for slow growth the new joints appear by division of the proximal phalanx rather than by addition of a phalanx at the distal tip. This show an intrinsic proximal phalanx plasticity that has never been observed in the real biological systems and it appear that any change in digit growth speed would uncover it. Is there any mutant where lower growth speed (E.g. lower Fgf signal) lead to splitting more proximal phalanges? They also don't provide enough details of how they are modeling growth. Are they just stretching the whole digit distally? In alternative, would a distal accretion growth make the system more robust to growth change?

Firstly, we should clarify how we are modelling growth in our simulations. As you suggest, apart from Fig 2D, we are only considering "distal accretion growth" (in which digits elongate at their distal tip by incorporation of sub-AER progenitors), and have modified Fig 2B to clarify this. As you point out, this distal accretion growth ensures that new joints form towards the distal end of the digit ray (for a wide range of growth rates), such that proximal phalanges do not split (Fig 2B).

We did find that changing the growth rate affected the precise dynamics in the distal region, with "fast growth" driving stripe formation at the very tip, and "slow growth" forming joints at the middle of the most distal phalanx. However, we realize that this description may have been confusing. In particular, in our original draft, we presented the two simulations as having "fast" or "slow" growth, relative to molecular patterning kinetics. The way we implemented this in our simulations was by changing the overall timescale of the simulations, but keeping the initial and final digit sizes the same. A longer overall timescale can be interpreted as either slower growth relative to patterning kinetics, or faster patterning kinetics relative to growth. We realize that, since digit growth can then be directly measured whereas the rate constants in our model are unknown, it makes more sense to explain the results assuming a constant growth rate and varying patterning kinetics. Therefore, "fast/slow growth" is better written as "slow/fast patterning kinetics". We have changed Figure S2A and lines 260-268 accordingly and hope that this makes more sense.

These simulations (Fig S2A) reveal that an intermediate range of growth rates can result in joint initiation at the distal tip. Without quantitative measurements, we cannot determine whether measured growth rates/patterning kinetics are consistent with our model predictions. However, our simulations demonstrate that it is certainly possible to specify joints distally within our model framework. Further work (including looking at mutants with different growth rates as you propose) would be another test of the model.

Finally, as previously discussed, we have explored alternative ways to prevent splitting of proximal phalanges, by (i) incorporating boundary effects from the PFR/DC (Fig. S2B) or (ii) explicitly forcing proximal cells to commit to joint/phalanx fate (Fig. 2C).

Minor point: They should avoid the use of the term "joint molecules" in the abstract as it can be confusing they can instead use "molecular joint markers".

This is a good suggestion and we have incorporated it into the abstract (lines 10).

© 2020. Published by The Company of Biologists under the terms of the Creative Commons Attribution License (https://creativecommons.org/licenses/by/4.0/).

Second decision letter

MS ID#: DEVELOP/2019/183699

MS TITLE: A dot-stripe model of joint patterning in the tetrapod limb

AUTHORS: Jake Cornwall Scoones and Tom Hiscock

I have now received all the referees reports on the above manuscript, and have reached a decision. The referees' comments are appended below, or you can access them online: please go to BenchPressand click on the 'Manuscripts with Decisions' queue in the Author Area.

The overall evaluation is very positive and we would like to publish your manuscript in Development, provided that the referees' remaining minor and purely editorial comments can be satisfactorily addressed. Please attend to these comments in your revised manuscript . If you do not agree with any of their criticisms or suggestions explain clearly why this is so.

Reviewer 1

Advance summary and potential significance to field

In this manuscript, Cornwall-Scoones and Hiscock present a “generic” model for periodic digit phalanx-joint patterning using two coupled Turing mechanisms to generate patterns of phalanx condensations (dots) and interzone joint progenitors (stripes). Using hypothetical Turing networks without specifying molecular components, they focus on how variant periodic patterns can be generated and modulated by altering boundary and growth conditions to recapitulate patterns for wrist bones/joints, which are more lattice-like (polygonal), for mutants with altered digit joint patterns, and for vertebrate taxa with highly modified phalanx/joint patterns. The ideas presented are interesting and will stimulate further experimental work to test the fit and utility of the model.

Comments for the author

The authors have extensively revised their manuscript based on reviewers' comments which has greatly improved the clarity and rationale used. They now demonstrate the superiority of the coupled 2-Turing system in modeling phalanx-joint formation, incorporate known developmental features and consider growth effects (phalanx formation from digit tip, progressive shortening of more distal phalanges), and define parameters in greater detail to make the math more accessible.

I have only one minor comment/suggestion. The term "intuition" is used in several places, and with different apparent meanings in mind than the standard definition. Perhaps other terms would be more appropriate?:

line 291 - "the intuition here is that once the dot spacing is lower than the digit width, then multiple dots can fit...." - prediction?

line 376 - "provide an intuition for why a combination of dot- and stripe-forming systems is required" - insight?

line 448 - "...we confirm this intuition with simulations in Fig. 2D." - proposal? prediction?

line 684 - " This confirms our intuition that dots are inhibiting stripes" - model? prediction?

Reviewer 3

Advance summary and potential significance to field

Cornwall and Hiscock have done a great job extending the theoretical analysis of the dot-stripe model and the main text. In table S1, they now present candidate genes for the stripe, dot and dot-

© 2020. Published by The Company of Biologists under the terms of the Creative Commons Attribution License (https://creativecommons.org/licenses/by/4.0/).

hole patterns considered in the model. In Figure 2C, they relate the model to phalanx forming regions (PFRs) and predict the minimal PFR size required by the model. In Figure 2D, they describe the type of growth considered in the model. In addition, they have developed an extended model that combines a PFR active region, distal growth and uniform growth. This model predicts that proximal phalanxes are longer providing an intriguing novel explanation for the smaller progressive size of distal phalanxes. Finally, in Figure 5, they compare the 4-species dot-stripe Turing model with a simple Activator-Inhibitor and a simple Substrate-Depleted model. Their analysis shows that although the Activator-Substrate Turing model can capture the overall arrangement of phalanx markers, it cannot generate narrow and straight stripe patterns. The analysis of the model is now comprehensive and it represents a valuable starting point to designing experiments to test if a Turing mechanism is involved in phalanx patterning. The paper has improved substantially and it is now suitable for publication.

Comments for the author

I have only a minor observation: why they have not included tenascin in table S1? It appears to be the gene that better fits the narrow stripe pattern generated by their model. Is it for the lack of joint phenotypes?

Finally, I have a suggestion about the title, it currently lacks any reference to the self-organizing/Turing nature of the model. Maybe they can change it to "A dot-stripe Turing model of joint patterning in the tetrapod limb".

Second revision

Author response to reviewers' comments

Reviewer 1 Comments for the Author:
The authors have extensively revised their manuscript based on reviewers' comments which has greatly improved the clarity and rationale used. They now demonstrate the superiority of the coupled 2-Turing system in modeling phalanx-joint formation, incorporate known developmental features and consider growth effects (phalanx formation from digit tip, progressive shortening of more distal phalanges), and define parameters in greater detail to make the math more accessible.

> We are pleased to hear that our revisions have improved the manuscript, and thank the reviewers for their useful comments.

I have only one minor comment/suggestion. The term "intuition" is used in several places, and with different apparent meanings in mind than the standard definition. Perhaps other terms would be more appropriate?:

line 291 - "the intuition here is that once the dot spacing is lower than the digit width, then multiple dots can fit...." - prediction?

> We have changed this to “prediction”.

line 376 - "provide an intuition for why a combination of dot- and stripe-forming systems is required" - insight?

> We have changed this to “insight”.

line 448 - "...we confirm this intuition with simulations in Fig. 2D." - proposal? prediction?

> We have changed this to “proposal”.

© 2020. Published by The Company of Biologists under the terms of the Creative Commons Attribution License (https://creativecommons.org/licenses/by/4.0/).

line 684 - " This confirms our intuition that dots are inhibiting stripes" - model? prediction?

We have changed this to "interpretation".

Reviewer 3 Advance Summary and Potential Significance to Field:
Cornwall and Hiscock have done a great job extending the theoretical analysis of the dot-stripe model and the main text. In table S1, they now present candidate genes for the stripe, dot and dot-hole patterns considered in the model. In Figure 2C, they relate the model to phalanx forming regions (PFRs) and predict the minimal PFR size required by the model. In Figure 2D, they describe the type of growth considered in the model. In addition, they have developed an extended model that combines a PFR active region, distal growth and uniform growth. This model predicts that proximal phalanxes are longer providing an intriguing novel explanation for the smaller progressive size of distal phalanxes. Finally, in Figure 5, they compare the 4-species dot-stripe Turing model with a simple Activator-Inhibitor and a simple Substrate-Depleted model. Their analysis shows that although the Activator-Substrate Turing model can capture the overall arrangement of phalanx markers, it cannot generate narrow and straight stripe patterns. The analysis of the model is now comprehensive and it represents a valuable starting point to designing experiments to test if a Turing mechanism is involved in phalanx patterning. The paper has improved substantially and it is now suitable for publication.

We are pleased to hear that our revisions have improved the manuscript, and thank the reviewers for their useful comments.

Reviewer 3 Comments for the Author:
I have only a minor observation: why they have not included tenascin in table S1? It appears to be the gene that better fits the narrow stripe pattern generated by their model. Is it for the lack of joint phenotypes?

Whilst tenascin does clearly show a stripe-like gene expression pattern, it is expressed after joint interzones have already formed (i.e. it is a "late" rather than an "early" marker). For this reason, and as suggested by Reviewer 1, we omitted tenascin from Table S1.

Finally, I have a suggestion about the title, it currently lacks any reference to the self-organizing/Turing nature of the model. Maybe they can change it to "A dot-stripe Turing model of joint patterning in the tetrapod limb".

Yes, this is an excellent suggestion, and we have changed the title accordingly.

Third decision letter

MS ID#: DEVELOP/2019/183699

MS TITLE: A dot-stripe Turing model of joint patterning in the tetrapod limb

AUTHORS: Jake Cornwall Scoones and Tom Hiscock
ARTICLE TYPE: Research Article

I have looked carefully at the revision of your manuscript and your answers to the few remaining reviewers'comments and in light of this I am happy to tell you that your manuscript has been accepted for publication in Development, pending our standard ethics checks.

© 2020. Published by The Company of Biologists under the terms of the Creative Commons Attribution License (https://creativecommons.org/licenses/by/4.0/).
